# A Structure–Activity Relationship Study of Alpha Synuclein PET Radiotracer M503-1619

**DOI:** 10.3390/molecules31142513

**Published:** 2026-07-18

**Authors:** Gui-Long Tian, Chia-Ju Hsieh, Dinahlee Saturnino Guarino, Zsofia Lengyel-Zhand, Wai Kit Chia, Shihong Li, Thomas J. A. Graham, Catherine Hou, Hsiaoju Lee, Anthony J. Young, E. James Petersson, Robert H. Mach

**Affiliations:** 1 Department of Radiology, Perelman School of Medicine, University of Pennsylvania, Philadelphia, PA 19104, USA; 2 Department of Chemistry, University of Pennsylvania, Philadelphia, PA 19104, USA

**Keywords:** alpha synuclein, structure–activity relationship, M503-1619, positron emission tomography, radiotracer

## Abstract

A previous study identified [^11^C]M503-1619 as a lead compound for positron emission tomography (PET) radiotracer development. The goal of the current study was to conduct a structure–activity relationship (SAR) study on M503-1619 to improve its in vitro binding affinity for alpha synuclein (aSyn), as well as to identify potential radiotracers that could be labeled with fluorine-18. The results of the SAR study identified strict SARs regarding the introduction of a fluorine into the *N*-aryl piperazine and benzamide moieties. The most promising compound was the corresponding 2-fluoroethyl analog of M503-1619. This compound was radiolabeled with fluorine-18, followed by in vivo PET imaging studies on non-human primate and in vitro autoradiography study. In vitro autoradiography studies in human postmortem brain sections demonstrated that ^18^F-labeled radiotracer has higher binding to synucleinopathies versus control brain tissues. This radiotracer displays the high initial brain uptake and rapid washout that is needed for a PET radiotracer for imaging a protein such as aSyn that has a low target density in the brain. However, the formation of brain penetrant radiolabeled metabolites prevented further evaluation of this compound. Insights from this SAR study are guiding the development of novel aSyn radioligands that avoid formation of the undesirable radiolabeled metabolite.

## 1. Introduction

The formation of insoluble aggregates of the protein, alpha synuclein (aSyn), is a neuropathological feature used in the postmortem characterization of a family of neurodegenerative disorders referred to as the synucleinopathies. The insoluble aggregates fall into two categories, the first being Lewy bodies and Lewy neurites, which are found in Parkinson’s disease (PD), dementia with Lewy bodies (DLB), and Parkinson’s disease with dementia (PDD) [1,2]. The second form of aSyn aggregates are glial cell inclusions (GCIs), which are found under two neurological conditions involving multiple system atrophy—cerebellar type (MSA-C) and Parkinsonian type (MSA-P) [3]. The development of radiotracers that can image insoluble aggregates of aSyn in vivo with positron emission tomography (PET) has proven to be quite challenging and has been limited by the number of compounds that bind to aSyn aggregates and not amyloid beta (Aβ) plaques, a copathology often found in PD, DLB and PDD [4,5].

Despite these limitations, a number of PET radiotracers displaying limited success in imaging aSyn in patients with PD and MSA have been recently reported [6,7,8,9,10]. For instance, the PET radioligand, [^18^F]ACI-12589, has shown its potential for detecting aSyn aggregations in patients with MSA, but not PD [6]. Another radiotracer, [^18^F]SPAL-T-06, demonstrated its capacity to image aSyn pathologies in both MSA-P and MSA-C, although its performance in PD remains unclear [11]. [^11^C]HY-2-15 was identified as a mixed aSyn and tau PET radiotracer [12]. However, results from a pilot clinical trial suggest that [^11^C]HY-2-15 is more favorable for imaging 4R tauopathies in patients with PSP than aSyn pathologies in patients with PD or MSA [13]. To date, only a few PET radioligands have shown potential in imaging aSyn aggregations in both patients with PD and MSA, including [^18^F]C05-05 [7]. In addition, a recent preliminary clinical result for [^11^C]MODAG-005 revealed its possibility of imaging aSyn pathologies in both PD and MSA patients [14], and a larger-scale study will be required to determine its clinical impact.

**Figure 1:**
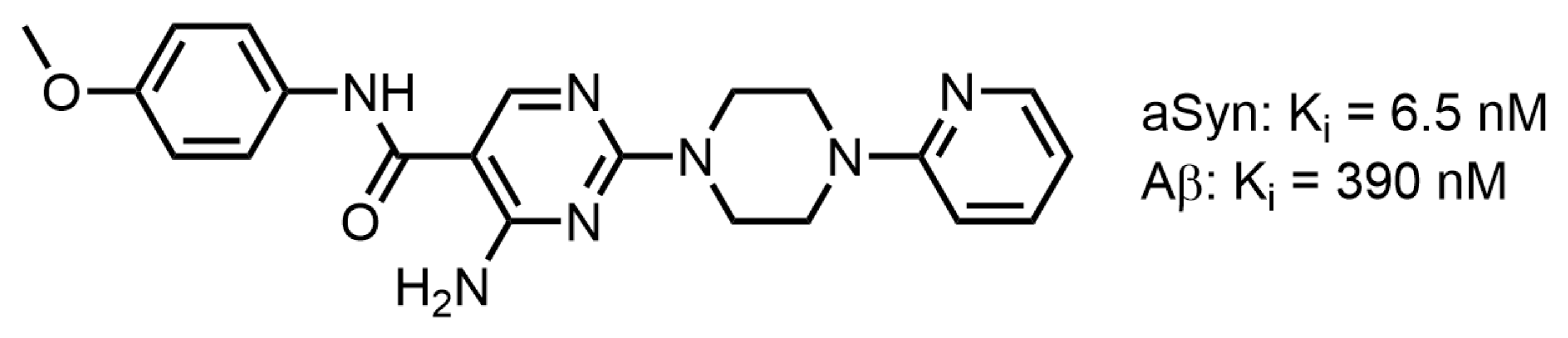
Structure and in vitro binding properties of M503-1619.

Our group previously reported the identification of a small molecule having a high affinity for aSyn and low affinity for Aβ using ultrahigh throughput in silico screening of a compound library consisting of 42 million compounds [15]. This compound, M503-1619 (Figure 1), was radiolabeled with tritium and was found to bind to aSyn in PD brains (Lewy bodies and neurites) but not MSA brains (GCIs) in an autoradiography study [15]. M503-1619 was further radiolabeled with carbon-11 and revealed favorable pharmacokinetic properties in non-human primate (NHP) PET imaging studies. As part of our continuing effort to develop a PET radiotracer for in vivo imaging of aSyn with PET, we conducted a detailed structure–activity relationship (SAR) study of M503-1619 with the goal of identifying a compound with an improved affinity for aSyn and selectivity versus Aβ, as well as to identify potential radiotracers that could be labeled with fluorine-18. This paper describes the results of this SAR study and provides the insights for developing novel aSyn PET tracers in this scaffold.

## 2. Results

### 2.1. Chemistry

**Scheme 1:**
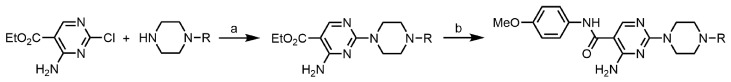
Synthesis of M503-1619 analogs. Conditions: a. Et_3_N, MeCN, rt., overnight; b. (1) NaOH, EtOH, 50 °C, 4 h; (2) Corresponding amines, HBTU, DIPEA, DMF, rt., overnight.

**Scheme 2:**
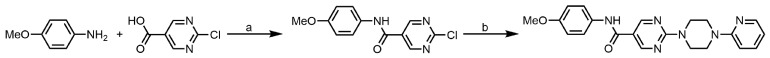
The method for the synthesis of **GT-5-121**. Conditions: a. SOCl_2_, DCM, 0 °C to rt., overnight; b. 1-(pyridin-2-yl)piperazine, MeCN, Et_3_N, rt. overnight.

**Scheme 3:**
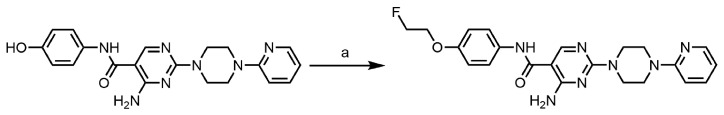
The method for the synthesis of **GT-2-114**. Conditions: 1-fluoro-2-iodoethane, Cs_2_CO_3_, DMF, 60 °C, 6 h.

The synthesis of the M503-1619 analogs is outlined in Scheme 1 and involves the coupling of ethyl 4-amino-2-chloropyrimidine-5-carboxylate with a panel of *N*-aryl piperazines to give ester intermediates in modest yield, followed by an ester hydrolysis and amide coupling yielding the analogs. A panel of compounds contained a fluorine group in the *N*-aryl piperazine fragment for potential radiolabeling with fluorine-18 (t_1/2_ = 109.8 min). **GT-5-121** was also synthesized to determine the importance of the amino group in the central pyrimidine ring upon aSyn binding (Scheme 2). Compounds containing a fluorine-, 2-fluoroethyl, or 3-fluoro-2-hydroxypropyl group in the benzamide moiety were also synthesized as an alternative strategy for PET radiolabeling with fluorine-18. The synthesis of these compounds can be found in Scheme 1, Scheme 2 and Scheme 3.

**Scheme 4:**
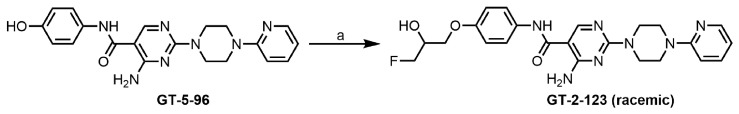
The methods for the synthesis of **GT-2-123**. Conditions: a. 2-(fluoromethyl)oxirane, Cs_2_CO_3_, DMF, 80 °C, 6 h.

The synthesis of the (+/−)-3-fluoro-2-hydroxypropyl analog was prepared by treating the phenol intermediate, **GT-5-96**, with 2-(fluoromethyl)oxirane under basic conditions (Scheme 4).

### 2.2. In Vitro Binding

The affinity of the target compounds for aSyn was performed in recombinant human fibrils using an in vitro radioligand binding assay with [^3^H]BF2846 as the radioligand, which binds to site 9 in recombinant aSyn fibrils [16]. The selectivity of the compounds for aSyn versus Aβ was measured via a counter screening assay in Alzheimer disease (AD) brain tissue homogenates using [^3^H]PiB as the radioligand. The results of the in vitro binding assay are shown in Table 1. K_i_ values are the average of N = 3.

The results of the in vitro binding assay revealed a very strict SAR regarding the *N*-aryl substituent of the piperazine ring (Table 1). For example, changing the 2-pyridyl ring of M503-1619 to a 4-pyridyl ring resulted in a reduction in binding affinity to aSyn (**GT-9-38**; K_i_ = 142 nM vs. 6.5 nM for M503-1619), whereas the corresponding 3-pyridyl analog was completely inactive in this assay (**GT-9-39**; K_i_ > 1000 nM). However, the introduction of a second nitrogen in the aromatic ring to give either the 2-pyrimidine (**GT-9-40**; K_i_ = 41.9 nM) or 2-pyrazine (**GT-9-41**; K_i_ = 13.5 nM) analog resulted in a relatively minor reduction in affinity relative to M503-1619. The introduction of an F-substituent into the *N*-2-pyridine moiety (**GT-9-63**, **GT-9-64**, **GT-9-67**, and **GT-9-68**) of M503-1619 resulted in a complete loss of binding to aSyn fibrils.

The addition of a second aromatic ring to the *N*-pyridyl group of M503-1619 resulted in either no change or a slight improvement in affinity for aSyn. The most potent analogs were **GT-5-84** and **GT-5-107**, which have an affinity for aSyn that was higher than M503-1619 (K_i_ = 1.6 nM for **GT-5-84** and 1.0 nM for **GT-5-107**). Both compounds had a low affinity for Aβ (K_i_ > 1000 nM).

The introduction of a fluorine or a fluorine-containing moiety in the benzamide aromatic ring generally led to modest reduction in affinity for aSyn (Table 2). An exception to this trend was the 2-fluoroethoxy analog, **GT-2-114**, which had an aSyn affinity of 4.9 nM and no measurable binding to Aβ. This compound was identified as a candidate for radiolabeling and in vivo imaging studies.

### 2.3. Radiosynthesis of ***[^18^F]GT-2-114***, PET Imaging in NHP and Autoradiography Studies in Postmortem Human Brain Sections

**Scheme 5:**
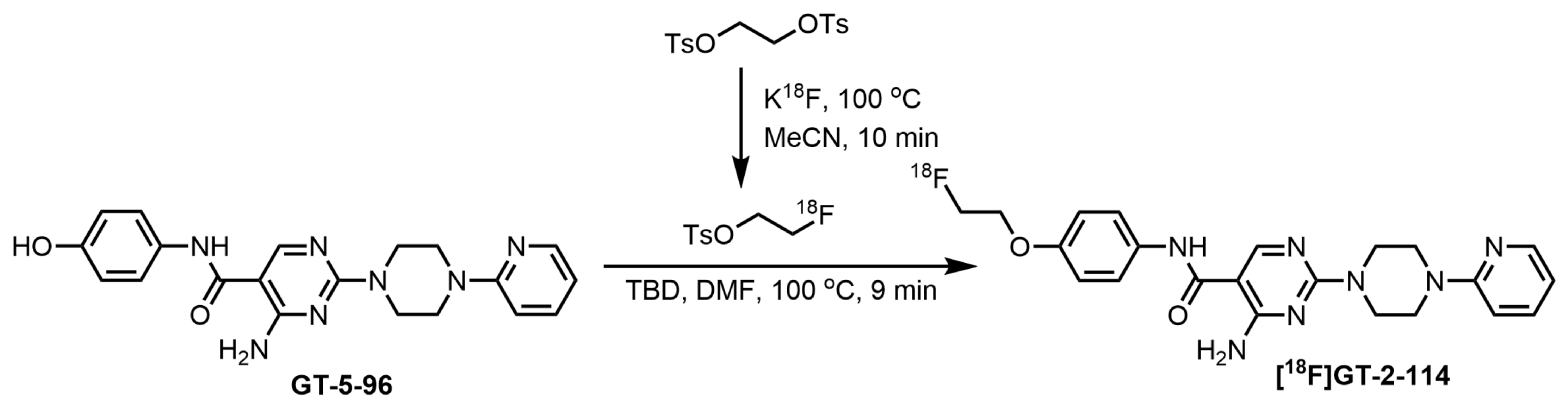
Radiosynthesis of **[^18^F]GT-2-114**.

The radiosynthesis of **[^18^F]GT-2-114** was accomplished via O-alkylation of the phenol precursor, **GT-5-96**, with [^18^F]2-fluoroethytosylate as outlined in Scheme 5. This is a two-step, two-pot reaction. The synthesis of [^18^F]2-fluoroethyltosylate ([^18^F]FEOTs) was accomplished following our previously published literature procedures [17]. Briefly, the formation of synthon, [^18^F]FEOTs, was accomplished by nucleophilic substitution. The excess of ditosylate was removed after the reaction. The fluorinated synthon was then reacted with hydroxyl precursor before the purification by a semi-prep HPLC. The detailed synthesis procedures are provided in the Section 4.3. The radiotracer was produced in a decay-corrected yield of 19.8% and specific activity was 130.94 GBq/µmol at the end of synthesis. The radiochemical purity was greater than 99.5%.

**Figure 2:**
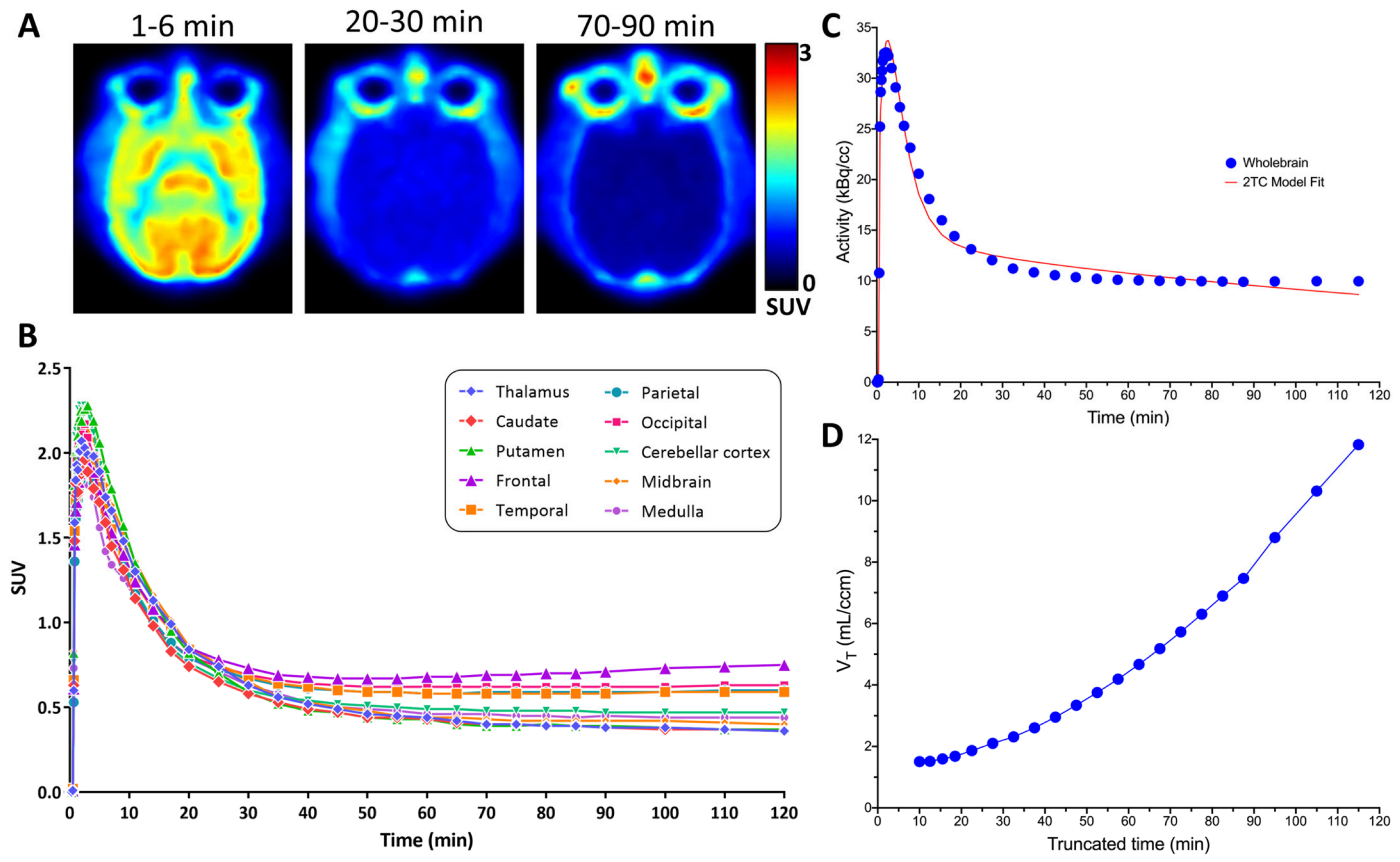
In vivo PET study of the female non-human primate for **[^18^F]GT-2-114**. (**A**) Representative brain images of 1–6 min, 20–30 min, and 70–90 min. (**B**) Time–activity curves of different brain regions. SUV, standardized uptake value. (**C**) Modeling of whole-brain **[^18^F]GT-2-114** activity. Fitting of whole-brain time–activity curve with a two-tissue compartmental model with radiometabolite-corrected plasma input function. (**D**) Time stability analysis of whole-brain V_T_. Estimates of the two-tissue compartment model V_T_ evaluated across truncated scan durations.

The **[^18^F]GT-2-114** NHP PET images showed high initial brain uptake, with a whole-brain peak standardized uptake value (SUV) of 1.82 (Figure S1) and 2.29 (Figure 2) occurring at around 1–3 min. The whole-brain peak-to-60 min ratio of SUV was 3.7 for the male NHP, and 3.9 for the female NHP. The rate of washout was slightly faster in the subcortical regions (peak-to-60 min ratio of SUV: 4.0 to 5.3) including thalamus, caudate, putamen, cerebellar cortex, midbrain, and medulla than in the cortical regions (peak-to-60 min ratio: 2.9 to 3.5), such as frontal, temporal, parietal, and occipital cortex (Figure 2B).

Metabolite analysis was conducted on venous blood samples and revealed that the metabolites were more polar than the parent compound (Figure S2 and Table S1). **[^18^F]GT-2-114** was rapidly metabolized, having a parent fraction of 18% by 30 min post-tracer injection. The free fraction of the radiotracer in plasma was 0.28%. The two-tissue compartmental model with radiometabolite-corrected plasma input function was used for further kinetic modeling. Fitting whole-brain activity to a two-tissue compartment model was poor, with large divergences between the model curve and tissue data (Figure 2C). Estimates of whole-brain V_T_ were also unstable, with a non-linear increase as scan duration increased (Figure 2D). Taken together, these findings and the late plateau in brain activity raise concern of increasing levels of non-specific binding, potentially from a blood–brain barrier penetrant radiometabolite.

In vitro autoradiography studies were performed in postmortem brain sections obtained from PDD, DLB, MSA-P and control. The results are shown in Figures S3 and S4 and show a higher binding of **[^18^F]GT-2-114** in the synucleinopathies brain sections including PDD, DLB and MSA-P versus control. There is a trend of higher binding to pathology ratio in Lewy body diseases (PDD and DLB) than in MSA-P (Figure S4B).

## 3. Discussion

Our group recently reported the in vitro and in vivo characterization of the aSyn radiotracer, [^11^C]M503-1619 [15]. This radiotracer was identified through an ultrahigh throughput in silico screening of a 42 million compound library using a “pseudostructure” or Exemplar for site 9 of the solid-state NMR structure of aSyn. Although the preliminary in vivo results were encouraging, this study concluded that additional SAR studies were needed to improve the aSyn affinity and imaging properties of [^11^C]M503-1619. The goal of the current study was to conduct this SAR study, with a special emphasis on identifying candidate PET radiotracers that could be radiolabeled with fluorine-18. The 110 min half-life of fluorine-18 has several advantages over the relatively short half-life of carbon-11 (t½ = 20.4 min).

The first structural modification involved exploring the substituent effects in the aromatic ring of the *N*-aryl piperazine moiety. Moving the position of the nitrogen of the pyridine ring from the 2-position in M503-1619 to the 3- or 4-position resulted in a dramatic reduction in affinity for aSyn. The addition of a second nitrogen to form the corresponding *N*-pyrimidine or *N*-pyrazole analog also resulted in a reduction in affinity relative to M503-1619. Surprisingly, the addition of a second aromatic ring to give the corresponding 2- or 3-quinoline or isoquinoline, or a 2-quinoxaline analog, led to an increase in affinity at aSyn at the expense of increasing the calculated log P of the compound to 3.7 to 4.3. Replacement of the 6-membered ring with a thiazole ring also led to a reduction in affinity at aSyn. These data indicate that the 2-pyridine is the preferred group at the *N*-aryl piperazine moiety.

Introduction of a fluorine into the different positions of the *N*-2-pyridine moiety resulted in a complete loss in affinity for aSyn. This reduction in affinity is likely due to the creation of an adverse stereoelectronic interaction with aSyn fibrils. These results demonstrate the rigid SAR requirements in this fragment of the molecule with its interaction with aSyn.

We next looked at the introduction of a fluorine group into the aromatic ring of the benzamide moiety of M503-1619. In most cases, introduction of a fluorine group directly onto the benzamide aromatic ring on M503-1619 led to a reduction in affinity for aSyn (i.e., **GT-9-36** and **GT-9-37**). An exception to this is when the methoxy group of M503-1619 was replaced with a fluorine group, which resulted in a minor reduction in affinity for aSyn (i.e., **GT-2-122**). Introduction of a trifluoromethyl moiety, either directly to the aromatic ring (i.e., **GT-2-146**) or by replacement of the hydrogen atoms of M503-1619 with fluorine atoms (**GT-2-149**) resulted in a large reduction in affinity for aSyn. On the other hand, conversion of the methoxy group of M503-1619 to the corresponding 2-fluoroethoxy moiety (**GT-2-114**) led to a compound having a similar affinity for aSyn as that of the parent structure. However, introduction of a 3-fluoro-2-hydroxypropyl moiety, a common fragment used in PET radiotracer design, resulted in a reduction in affinity for aSyn.

The results of the above SAR study indicate that **GT-2-114** is the best-in-series compound resulting from this SAR study. Consequently, **[^18^F]GT-2-114** was radiolabeled using the [^18^F]2-fluoroethyltosylate as the radiolabeled synthon, and imaging studies were conducted in NHPs. **[^18^F]GT-2-114** had a high initial brain uptake (SUV > 2.0) and rapid washout, resulting in a peak to 60 min ratio of 3.29. The high affinity and rapid washout of **[^18^F]GT-2-114** from healthy NHP brain indicates that it has the potential to image aSyn aggregates in patients with Lewy body disease, which is typically expressed in lower density than the other insoluble protein aggregates in neurodegenerative disorders such as Aβ and tau in AD. Of course, further studies on animal models with aSyn pathologies will be needed to validate the capability of **[^18^F]GT-2-114** in imaging aSyn aggregations in vivo.

**[^18^F]GT-2-114** is also rapidly metabolized into compounds more polar than the parent. However, a trend of slightly increasing uptake in the cortical regions after 60 min post-tracer injection was observed in the NHP studies. In addition, attempts to estimate brain V_T_ were hindered by poor fitting of a two-tissue compartmental model of reversible binding and a divergence in estimates observed as scan duration increased. Both the trend of rising time–activity curves and difficulty in kinetic modeling indicated the potential blood–brain penetrant radiometabolites. Therefore, future study will likely benefit by considering the labeling position and compound structure to avoid generation of any similar potentially confounding radiometabolites.

A factor limiting the metabolism data is the relatively wide peaks observed around the retention time of the parent compound. We expect that this is primarily the broadening of the parent compound peak, based on prior testing of [^11^C]M503-1619 (unpublished data). However, we did not directly test this in **[^18^F]GT-2-114** or verify whether the parent peaks are free of potential radiometabolites with similar retention times. As compartmental modeling with blood inputs was unsuccessful, we did not pursue further testing to address the limitations presented by the lower peak resolution.

The in vitro autoradiography studies revealed that **[^18^F]GT-2-114** has higher binding to the postmortem brain sections of PDD, DLB and MSA-P than the control brain tissue. The brain sections of PDD, DLB and MSA-P were pathologically confirmed with aSyn aggregations and no Aβ, suggesting that **[^18^F]GT-2-114** may be a useful PET radiotracer for imaging aSyn aggregations in the brain. In addition, there was the observation of higher binding-to-pathology ratio in Lewy body diseases (PDD and DLB) than in MSA-P, indicating **[^18^F]GT-2-114** may preferentially bind to aSyn in Lewy bodies over GCIs in MSA-P. Nevertheless, the current assessment was based on a small sample size, involving two DLB brain sections containing tau pathology, and the lack of the tau screening assay was performed in this study. Therefore, the possibility of **[^18^F]GT-2-114** binding to tau aggregations could not be ruled out and the selectivity of aSyn over tau remains unknown.

In summary, this paper describes a SAR study aimed at generating a potential fluorine-18 analog of M503-1619, a PET radiotracer for imaging aSyn aggregates that was previously labeled with carbon-11. The results of this SAR study provided useful information on positions in this scaffold where the introduction of a fluorine is tolerated versus positions where a reduction in aSyn affinity was noted. Although a potential ^18^F-labeled candidate was identified, the formation of a radiolabeled metabolite that enters the brain prevented further evaluation of this compound. The results of this study are being incorporated into the design of aSyn radioligands that avoids this adverse radiolabeled metabolite.

## 4. Materials and Methods

### 4.1. Chemistry

All reagents and solvents for synthesis were purchased and used without further purification. Structures of synthesized chemicals were identified using ^1^H and ^13^C nuclear magnetic resonance (NMR) spectra, as well as mass spectroscopy. ^1^H and ^13^C NMR were recorded in ppm units relative to deuterated solvent (CDCl_3_) as an internal reference by Bruker DMX 500 MHz NMR instrument (Bruker, Billerica, MA, USA). ^1^H chemical shifts are reported in parts per million (ppm) and measured relative to tetramethylsilane (TMS). Mass spectra were acquired using a 2695 Alliance LC/MS (Waters Corporation, Milford, MA, USA). Purification of synthesized chemicals was conducted on Biotage Isolera One (Biotage, Salem, NH, USA) with a dual-wavelength UV–VIS detector. The synthesis and characterization of the compounds are described in Supplementary Materials (Chemical characterization of intermediates and final compounds).

### 4.2. In Vitro Binding Assay

In vitro radioligand biding assays were performed by using the pervious published method [12,15] to measure the affinity of the target compounds for aSyn and Aβ. In brief, the radioligand for the aSyn assay was [^3^H]BF2846 and the assay was conducted using recombinant fibrils. Three concentrations of test compounds (10 nM, 100 nM and 1 µM) were incubated with ~4 nM [^3^H]BF2846 and 50 nM aSyn fibrils in a working buffer (50 mM Tris-HCl, 0.01% bovine serum albumin) at 37 °C for 1.5 h. The mixture (150 µL) was separated by filtration through a Unifilter-96 system (PerkinElmer, Shelton, CT, USA) and washed with an ice-cold buffer (10 mM Tris-HCl (pH 7.4), 15 mM NaCl and 20% EtOH) before scintillation counting. Total binding was defined in the absence of competitor, and non-specific binding was determined using 100 nM unlabeled BF2846. For the full competition curves, [^3^H]BF2846 (~4 nM) was incubated with 50 nM aSyn fibrils and ten concentrations of test compounds (0.05 to 1000 nM). Data from three independent experiments were analyzed by non-linear regression in GraphPad Prism v.9.3.1 (GraphPad Inc., San Diego, CA, USA) to calculate K_i_ values using the equation, K_i_ = EC_50_/(1 + [radioligand]/K_d_).

The radioligand assay for Aβ was [^3^H]PiB. Three concentrations of test compounds (10 nM, 100 nM and 1 µM) were incubated with ~32 nM [^3^H]PiB and 0.5 µg/µL of AD, as well as CAA− tissue homogenate in Dulbecco’s phosphate-buffered saline (DPBS) at 37 °C for 1.5 h. Non-specific binding was defined by 1 µM of unlabeled PiB in DPBS. The following procedures were performed in the same manner as the aSyn assay, using DPBS instead of the working buffer.

### 4.3. Radiosynthesis of ***[^18^F]GT-2-114***

The radiosynthesis of **[^18^F]GT-2-114** was accomplished via O-alkylation of the phenol precursor, **GT-5-96**, with [^18^F]2-fluoroethytosylate, as outlined in Scheme 5. After trapping [^18^F]F^−^ from an Accell^®^ Light QMA Carbonate cartridge (Waters Corporation, Milford, MA, USA), the radioactivity was eluted with 1 mL eluent containing 0.85 mL MeCN, 0.15 mL H_2_O, 7 mg K222 and 1 mg K_2_CO_3_. After azeotropically drying with vacuum and heat (100 °C) several times in the first reaction vessel, an aliquot of 5 mg ethylene di(*p*-toluenesulfonate) in 1 mL MeCN was added. The reaction was carried out at 100 °C for 10 min and then cooled to 60 °C. After the completion of fluorination, 20 mL water was introduced into the first reaction vessel and the reaction mixture was then passed through a 33 mm 0.45 µm nylon filter to remove excess ditosylate precursor, and a fluorinated synthon, [^18^F]2-fluoroethytosylate ([^18^F]FEOTs), was concentrated on an OASIS Light HLB cartridge (Waters Corporation, Milford, MA, USA). The cartridge was washed with additional 10 mL of water. The [^18^F]FEOTs was eluted with 2 mL MeCN through a Sep-Pak Dry cartridge (Waters Corporation, Milford, MA, USA) into the second reaction vessel. The second step was carried out by adding 0.8 mL DMF containing 4 mg phenol precursor and 7 mg 1,5,7-triazabicyclo [4.4.0]dec-5-ene (TBD) into the second reaction vessel. The mixture was heated at 100 °C for 9 min and cooled down to 40 °C. The reaction was quenched with mobile phase before being loaded to HPLC loop for purification. A semi-preparative HPLC with an Agilent Zorbax SB-C18 column (Santa Clara, CA, USA) (5 µm, 100 × 9.4 mm) was used. The mobile phase contained 52% methanol in 20 mM ammonium bicarbonate solution and flow rate was 5 mL/min. The desired product was eluted around 22 min and the fraction was collected in a 20 mL syringe and then diluted with 15 mL water. The product was concentrated on a tC18 Light cartridge (Waters Corporation, Milford, MA, USA). The final product was eluted with 1 mL ethanol and passed through a 0.2 µm sterile Millex^®^ FG filter (Merck, Darmstadt, Germany) into final production vial. Afterward, 10 mL saline was also added. The analytical system was a Waters Acquity UPLC. The composition of mobile phase was 15% acetonitrile and 85% water containing 0.1% trifluoroacetic acid. A Waters BEH C18 column (1.7 µm; 2.1 × 50 mm) was used and the flow rate was 0.5 mL/min.

The decay-corrected yield was 19.8% and specific activity was 130.94 GBq/µmol at end of synthesis. The radiochemical purity is greater than 99.5%.

### 4.4. Radiometabolism Study of ***[^18^F]GT-2-114***

Radiometabolite analysis was performed on venous blood samples to determine the in vivo rate of metabolism of **[^18^F]GT-2-114**, with methods matching the analysis of [^11^C]M503-1619 as described by Tian et al. [15]. In brief, samples were centrifuged to separate plasma (1 mL), and plasma were then deproteinated with acetonitrile at 1:1 by volume. **[^18^F]GT-2-114** parent compound and radiometabolites were separated from deproteinated plasma supernatant using radioHPLC on an Agilent Infinity 1280 HPLC with a reverse-phase Agilent Zorbax XDB-C18 analytical HPLC column (4.6 × 150 mm) and a mobile phase of 65% 0.1 M ammonium formate buffer and 35% acetonitrile at 1.2 mL/min. Parent and metabolite activity fractions were measured with an in-line Posi-RAM detector (LabLogic Systems, Chantilly, VA, USA) based on retention times of **GT-2-114** standards.

### 4.5. PET Studies of ***[^18^F]GT-2-114*** in NHPs

The NHP study was performed under protocols approved by the University of Pennsylvania Institutional Animal Care and Use Committee (IACUC). Dynamic **[^18^F]GT-2-114** total-body PET/CT scans were conducted on a male (6-year-old, 10.6 kg) and a female (9-year-old, 6.5 kg) healthy rhesus macaques using the PennPET Explorer scanner (University of Pennsylvania, Philadelphia, PA, USA). Each NHP, fasted for 12 h prior to the PET study, was initially anesthetized by intramuscular injection with ketamine (4 mg/kg) and dexmedetomidine (0.05 mg/kg). Then, the NHP was intubated and anesthesia was maintained with 0.75–2% isoflurane/oxygen. A percutaneous catheter was placed for the tracer injection. Venous blood samples were obtained and processed as described by Tian et al. [15] to measure in vivo tracer metabolism. A low-dose CT scan was performed to confirm positioning and attenuation correction followed by 90 min (12 × 10 s, 2 × 30 s, 4 × 60 s, 2 × 120 s, 3 × 180 s, and 14 × 300 s) or 120 min (12 × 10 s, 2 × 30 s, 4 × 60 s, 2 × 120 s, 3 × 180 s, 14 × 300 s and 3 × 600 s) dynamic PET image acquired in list mode after venous injection of 102.1 (1 mL, male NHP) and 111.9 (1.25 mL, female NHP) MBq of **[^18^F]GT-2-114**. PET images were reconstructed using a time-of-flight list-mode ordered subsets expectation maximization (OSEM, 25 subsets) reconstruction algorithm [18]. The PET/CT data were analyzed by using Pmod software (version 3.7, PMOD Technologies Ltd., Zurich, Switzerland). Each perfusion phase (1–11 min) of brain PET image was co-registered to the corresponding MR-T1 weighted brain image. The individual MR-T1 weighted brain image was spatially normalized to the INIA19 rhesus macaque MR brain template [19]. Then, the spatial normalization parameters were applied to the corresponding dynamic brain PET image to form a spatially normalized brain PET image in the INIA19 template domain. Eleven volumes of interest (VOIs) modified from the INIA19 macaque brain atlas, including thalamus, caudate, putamen, frontal, temporal, occipital, parietal, midbrain, medulla, cerebellar cortex, and whole-brain, were used for further PET neuroimaging analyses. Time–activity curves were extracted from all the VOIs and expressed as SUV. The peak-to-60 min ratio was calculated as the SUV at the time of maximal uptake divided by the SUV at 60 min for each region.

To evaluate the feasibility of pharmacokinetic modeling, a whole-brain time–activity curve was fitted to a reversible two-tissue compartment model, and estimates of the total volume of distribution (V_T_) were recorded. The plasma input function was generated from whole-blood activity in a left ventricle VOI fitted to a tri-exponential function, corrected for plasma:whole-blood partitioning and radiometabolites based on sampled venous blood. Plasma:whole-blood partitioning was based on a linear-interpolation of the activity ratio of 200 µL aliquots of plasma and whole-blood from each venous sample. Radiometabolite corrections were calculated using a sigmoid function fit to the parent fraction at each sample time point. The two-tissue compartment model was first fit, with calculated blood delays and a blood volume fraction of 0.05, to data from the full scan duration. To determine the stability of V_T_ estimates with scan duration, the model was also fit to truncated whole-brain time–activity curves.

### 4.6. Human Brain Samples

Formalin-fixed paraffin-embedded (FFPE) human brain tissue from PDD, DLB, MSA and control cases were acquired from the Center for Neurodegenerative Disease Research (CNDR) at the University of Pennsylvania. Patients’ demographic data are presented in Table S2. A neuropathological diagnosis was obtained after review by a board-certified neuropathologist according to consensus criteria [20]. Neuropathological characterization was performed by immunohistochemistry using antibodies against tau (mouse antibody PHF1, a gift from Dr Peter Davies), Aβ (mouse antibody NAB228, generated in CNDR), aSyn (mouse antibody Syn303, generated at the CNDR) and TDP-43 (rat antibody 1D3, a gift from Elisabeth Kremmer and Dr Manuela Neumann) as previously described by the CNDR. Primary antibody binding was visualized using an avidin–biotin complex detection method with diaminobenzidine as the chromogen. Each region was assigned a semiquantitative pathology score by a board-certified neuropathologist: none (0), rare (0.5), mild (1), moderate (2), or severe (3), representing increasing pathological burden within the examined region.

### 4.7. ***[^18^F]GT-2-114*** In Vitro Real-Time Autoradiography

In vitro autoradiography was performed using 6 μm thick deparaffinized sections derived from MSA, DLB, PDD and control brains. Brain sections were first equilibrated for 30 min in 1× PBS (Dulbecco’s phosphate-buffered saline) and then incubated for 1 h at room temperature with 7 nM **[^18^F]GT-2-114** (specific activity 994.75 GBq/µmol at end-of-synthesis). The incubation solution was prepared by diluting 69.9 MBq of **[^18^F]GT-2-114** to a final volume of 10 mL, resulting in a final tracer concentration of approximately 7 nM and a radioactivity concentration of 6.99 MBq/mL. The sections were rinsed three times in cold buffer 1× PBS for 3 min, followed by a quick dip in cold distillated water. Non-specific binding was determined using 1.5 µM unlabeled **GT-2-114**. Slides were then allowed to air-dry before being exposed and scanned in a real-time autoradiography system (BeaQuant instrument, ai4R, Nantes, France) for 10h. Region of interest delimitation and quantification of signal was performed by using the image analysis software Beamage (version 4.0.3, ai4R, Nantes, France). Specific binding was determined by subtracting the non-specific signal from the total signal and expressed as counts/min/mm^2^. The ratio of specific signal in diseased versus control tissues was determined by dividing the specific signal in the diseased tissue over the average (if several cases were tested) specific signal in the control tissue for each brain region.

## 5. Conclusions

**[^18^F]GT-2-114** was identified following a SAR study on M503-1619, a compound initially identified via ultrahigh throughput in silico screening of a 42 million compound library. The compound, **GT-2-114**, exhibits promising in vitro properties including high affinity for aSyn, low affinity for Aβ, and ability to be radiolabeled with fluorine-18. The fluorine-18 labeled compound, **[^18^F]GT-2-114**, demonstrated a higher binding in aSyn pathologies in Lewy bodies compared with control postmortem brain tissues in autoradiography study. However, in vivo pharmacokinetics and metabolite NHP studies revealed the formation of a brain penetrant radiolabeled metabolite. This limitation prevented further evaluation and advancement of **[^18^F]GT-2-114** as a candidate PET radioligand. The results of this SAR study are being incorporated into the design of new aSyn radioligands that avoid this adverse radiolabeled metabolite.

## 6. Patents

A US patent application has been filed on the compounds reported in this publication.

## Figures and Tables

**Table 1:** SAR at the *N*-aryl piperazine group.

Compound #	Structure	[^3^H]BF2846 in aSyn Fibrils (K_i_; nM)	[^3^H]PiB in AD Brain Tissue (K_i_; nM)	Aβ-to-aSyn Ratio	cLogP ^a^
**GT-5-83**	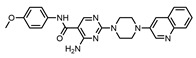	4.4 ± 4.2	>1000	>227.3	4.27
**GT-5-84**	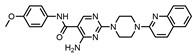	1.6 ± 0.4	>1000	>625.0	4.27
**GT-5-93**	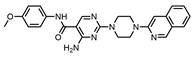	7.7 ± 4.6	>1000	>129.9	4.06
**GT-5-105**	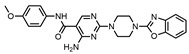	6.0 ± 3.1	>1000	>166.7	3.66
**GT-5-107**	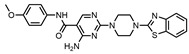	1.0 ± 1.6	>1000	>1000.0	4.37
**GT-5-118**	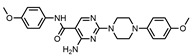	44.8 ± 2.8	>1000	>22.3	3.86
**GT-5-119**	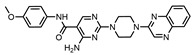	6.9 ± 4.6	23.0 ± 91.0	3.3	3.72
**GT-5-121**	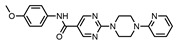	6.8 ± 2.6	147.0 ± 232.0	21.6	2.29
**GT-9-38**	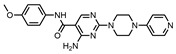	142.3 ± 65.9	Not detected	>7.0	2.90
**GT-9-39**	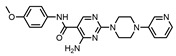	>1000	Not detected	-	2.89
**GT-9-40**	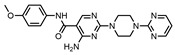	41.9 ± 13.8	Not detected	>23.9	2.12
**GT-9-41**	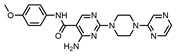	13.5 ± 8.3	297.2 ± 326.0	22.0	2.12
**GT-9-42**	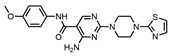	29.4 ± 2.7	>1000	>34.0	2.17
**GT-9-63**	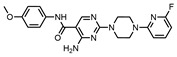	Not detected	Not detected	-	3.12
**GT-9-64**	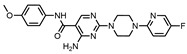	Not detected	Not detected	-	3.12
**GT-9-67**	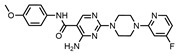	Not detected	Not detected	-	3.12
**GT-9-68**	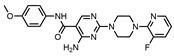	Not detected	Not detected	-	3.12

Data is presented as mean ± standard deviation. Not detected: compounds with low displacement of [^3^H]BF2846 or [^3^H]PiB in the initial inhibition screening assay. ^a^ Calculated using ChemDraw Professional 23.1.

**Table 2:** SAR at the benzamide moiety.

Compound #	Structure	[^3^H]BF2846 in aSyn Fibrils (K_i_; nM)	[^3^H]PiB in AD Brain Tissue (K_i_; nM)	Aβ-to-aSyn Ratio	cLogP ^a^
**GT-2-114**	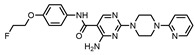	4.9 ± 1.5	Not detected	>204.1	3.14
**GT-2-122**	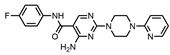	10.0 ± 0.3	Not detected	>100.0	3.12
**GT-2-123**	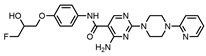	27.6 ± 6.8	Not detected	>36.2	2.20
**GT-2-146**	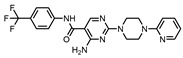	>1000	Not detected	-	3.89
**GT-2-149**	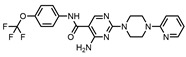	>1000	Not detected	-	4.00
**GT-9-36**	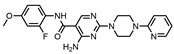	51.7 ± 27.0	>1000	>19.3	2.49
**GT-9-37**	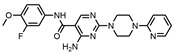	33.3 ± 11.6	275.4 ± 205.0	8.3	2.89

Data is presented as mean ± standard deviation. Not detected: compounds with low displacement of [^3^H]BF2846 or [^3^H]PiB in the initial inhibition screening assay. ^a^ Calculated using ChemDraw Professional 23.1.

## Data Availability

The original contributions presented in this study are included in the article/Supplementary Materials. Further inquiries can be directed to the corresponding author.

## Supplementary Materials

The following supporting information can be downloaded at: https://www.mdpi.com/article/10.3390/molecules31142513/s1. Figure S1: **[^18^F]GT-2-114** time–activity curves of different brain regions for the male non-human primate. Figure S2: HPLC chromatograms of a sample from (A) the dose vial and venous metabolites analyzed at (B) 7, (C) 17 and (D) 29 minutes post-i.v. injection of the radiotracer. Figure S3: Autoradiogram showing **[^18^F]GT-2-114** total binding (upper panel; determined with 7nM) and non-specific binding (lower panel; determined by blocking with 1.5μM of unlabeled **GT-2-114**) of FFPE postmortem brain sections from PDD, DLB, and MSA-P patients and CT. Autoradiography color/brightness levels are expressed in counts (0-100). Neuropathology scores for aSyn, Aβ, tau and TDP-43 are reported for each case. FFPE, formalin fixed paraffin embedded; PDD, Parkinson’s disease with dementia; DLB, Dementia with Lewy bodies; MSA-P, Multiple System Atrophy – Parkinsonian type; CT, control; aSyn, alpha synuclein; Aβ, amyloid beta; TDP-43, TAR DNA-binding protein 43. Figure S4: (A) Data shows **[^18^F]GT-2-114** specific binding in gray matter presented as counts/min/mm^2^ in CT, MSA-P, and the Lewy body diseases, PDD and DLB. Each bar represents an individual case. (B) Data presents the ratios of specific binding in the Lewy body diseases (blue; 2 DLB cases and 1 PDD case) and MSA-P (red) pathology over the brain region matched CT value. Data of LBD is presented as mean and standard deviation. PDD, Parkinson’s disease with dementia; DLB, Dementia with Lewy bodies; LBD, Lewy body disease; MSA-P, Multiple System Atrophy–Parkinsonian type; CT, control; GM, gray matter. Table S1: **[^18^F]GT-2-114** Parent fraction in female non-human primate. Table S2: Selected demographics of cases used for autoradiography. Chemical characterization of intermediates and final compounds.

## Abbreviations

The following abbreviations are used in this manuscript:

PD	Parkinson’s disease
PDD	Parkinson’s disease with dementia
DLB	Dementia with Lewy bodies
GCIs	Glial cell inclusions
MSA-C	Multiple System Atrophy—cerebellar type
MSA-P	Multiple System Atrophy—Parkinsonian type
AD	Alzheimer’s disease
Aβ	Amyloid beta
PET	Positron Emission Tomography
SAR	Structure–Activity Relationships
aSyn	Alpha synuclein
TDP-43	TAR DNA-binding protein 43
NHP	Non-human primate
SUV	Standardized uptake value
CT	Control
NMR	Nuclear magnetic resonance
VOI	Volumes of interest
FFPE	Formalin-fixed paraffin-embedded
CNDR	Center for Neurodegenerative Disease Research
