# Supplementary material for: A Structure–Activity Relationship Study of Alpha Synuclein PET Radiotracer M503-1619
Source: Molecules. 2026 Jul 18;31(14):2513. doi: 10.3390/molecules31142513 (PMC13416357; doi:10.3390/molecules31142513)
Supplement: Supplementary file 1 [file molecules-31-02513-s001.zip › molecules-4377628-supplementary.pdf]

Table of Contents

Figure S1. **[18F]GT-2-114** TACs of different brain regions for the male non-human primate 2

Figure S2. HPLC chromatograms of venous blood samples of **[18F]GT-2-114** 2

Figure S3. Autoradiogram of **[18F]GT-2-114** of postmortem brain sections 3

Figure S4. Quantitation of **[18F]GT-2-114** autoradiography in postmortem brain sections 3

Table S1. Parent fraction of **[18F]GT-2-114** in a female non-human primate 4

Table S2. Demographics of the cases used for autoradiography 4

Chemical characterization of intermediates and final compounds 5

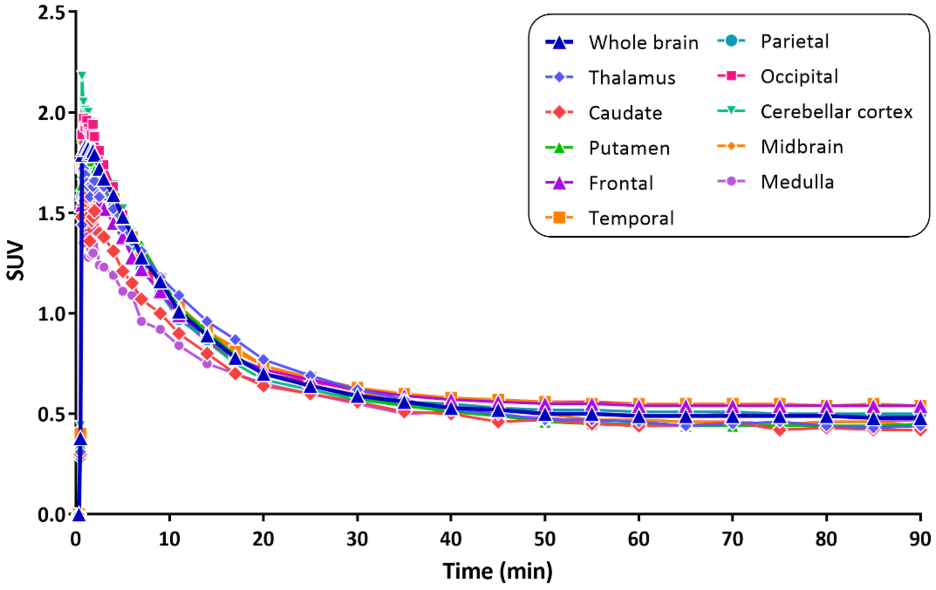

Figure S1. [18F]GT-2-114 time-activity curves of different brain regions for the male non-human primate.

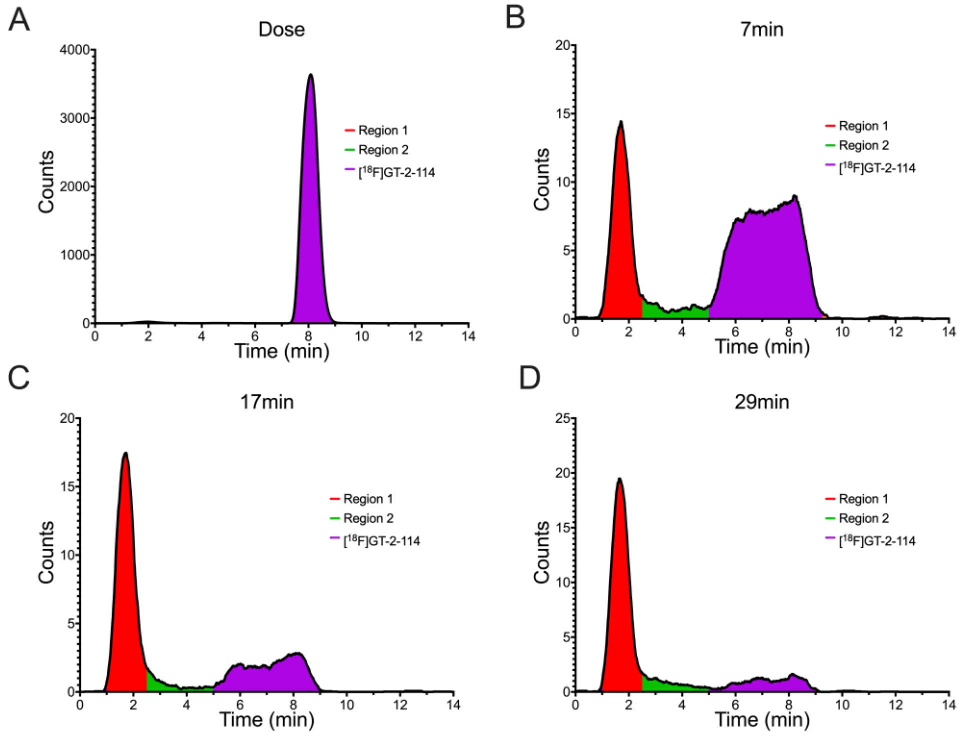

Figure S2. HPLC chromatograms of a sample from (A) the dose vial and venous metabolites analyzed at (B) 7, (C) 17 and (D) 29 minutes post-i.v. injection of the radiotracer.

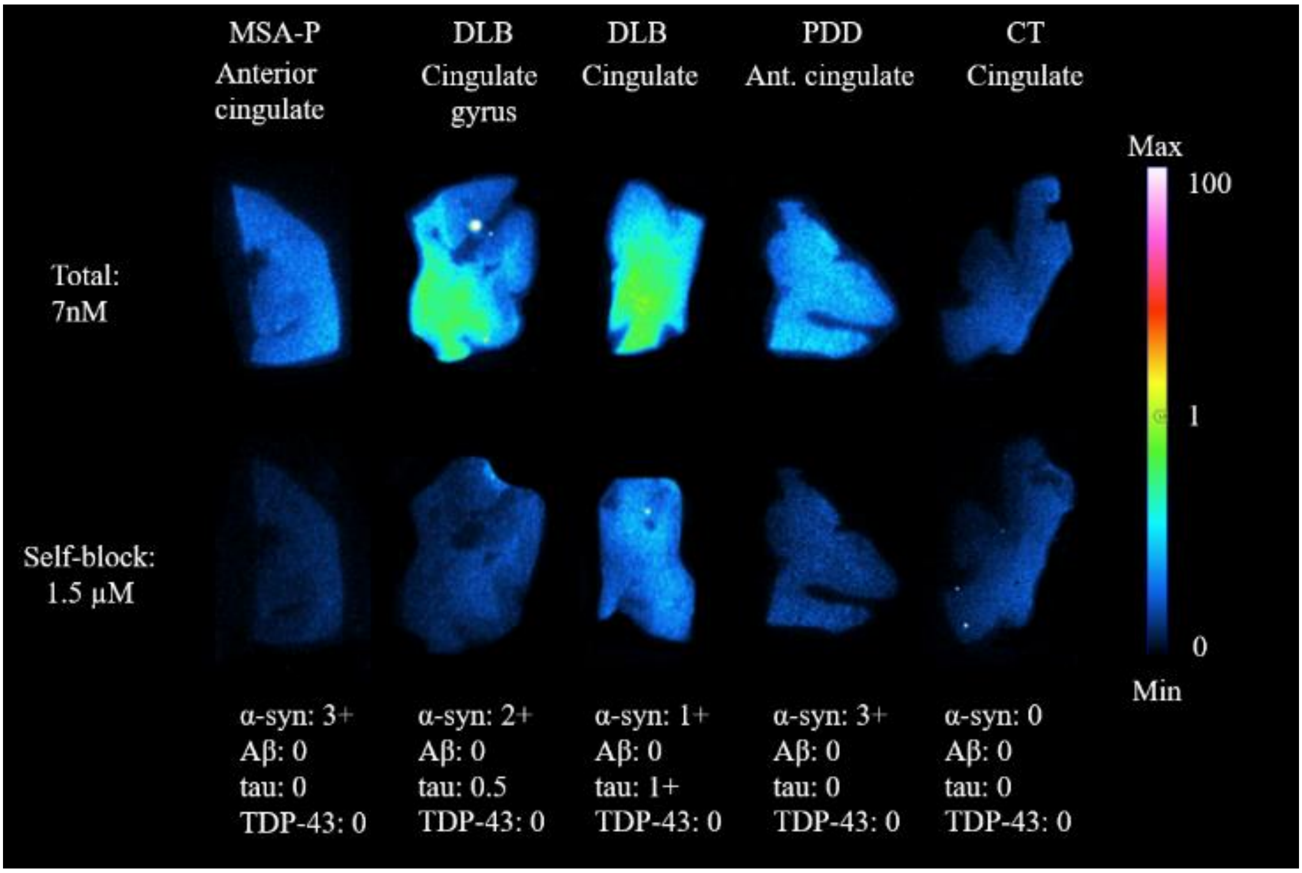

Figure S3. Autoradiogram showing **[18F]GT-2-114** total binding (upper panel; determined with 7nM) and non-specific binding (lower panel; determined by blocking with 1.5µM of unlabeled **GT-2-114**) of FFPE postmortem brain sections from PDD, DLB, and MSA-P patients and CT. Autoradiography color/brightness levels are expressed in counts (0-100). Neuropathology scores for aSyn, Aβ, tau and TDP-43 are reported for each case. FFPE, formalin fixed paraffin embedded; PDD, Parkinson's disease with dementia; DLB, Dementia with Lewy bodies; MSA-P, Multiple System Atrophy – Parkinsonian type; CT, control; aSyn, alpha synuclein; Aβ, amyloid beta; TDP-43, TAR DNA-binding protein 43.

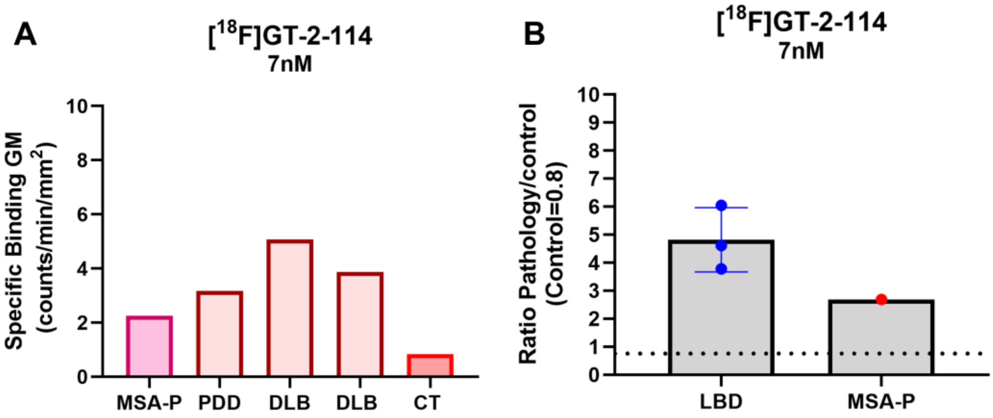

Figure S4. (A) Data shows [18F]GT-2-114 specific binding in gray matter presented as counts/min/mm² in CT, MSA-P, and the Lewy body diseases, PDD and DLB. Each bar represents an individual case. **(B)** Data presents the ratios of specific binding in the Lewy body diseases (blue; 2 DLB cases and 1 PDD case) and MSA-P (red) pathology over the brain region matched CT value. Data of LBD is presented as mean and standard deviation**.** PDD, Parkinson's disease with dementia; DLB, Dementia with Lewy bodies; LBD, Lewy body disease; MSA-P, Multiple System Atrophy – Parkinsonian type; CT, control; GM, gray matter.

Table S1. [18F]GT-2-114 Parent fraction in female non-human primate

Time post-injection (min)	Parent Fraction 1st Replicate (%)	Parent Fraction 2nd Replicate (%)
Dose	99%	-
6.7	65%	67%
17.3	28%	32%
29.4	19%	17%

Table S2. Selected demographics of cases used for autoradiography.

Case patient /control (P)/(C)	Gender (M/F)	Age of Onset (years)	Age of Death (years)	Anatomical Region	Diagnosis	α-syn	Aβ	tau	TDP43
P	F	41	56	Anterior cingulate	MSA-P	3+	0	0	0
P	M	51	59	Cingulate gyrus	DLB	2+	0	0.5	0
P	M	65	83	Cingulate	DLB	1+	0	1	0
P	M	49	72	Anterior cingulate	PDD	3+	0	0	0
C	F		60	Cingulate	Control	0	0	0	0

https://doi.org/10.3390/xxxxx

Characterization of Synthetic Intermediates and Final Compounds

All reagents and solvents for synthesis were purchased and used without further purification. Structures of synthesized chemicals were identified using ^{1}H and ^{13}C nuclear magnetic resonance (NMR) spectra, and mass spectroscopy. ^{1}H and ^{13}C NMR were recorded in ppm units relative to deuterated solvent ($CDCl_3$) as an internal reference by Bruker DMX 500 MHz NMR instrument (Bruker, Billerica, MA, USA). ^{1}H chemical shifts are reported in parts per million (ppm) and measured relative to tetramethylsilane (TMS). Mass spectra were acquired using a 2695 Alliance LC/MS (Milford, MA, USA). Purification of synthesized chemicals was conducted on Biotage Isolera One (Biotage, Salem, NH, USA) with a dual-wavelength UV-VIS detector.

General procedure for the synthesis of M503-1619 analogs

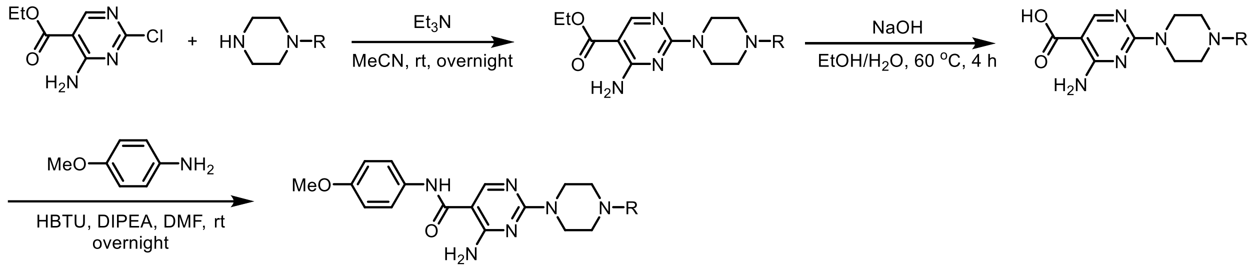

Ethyl 4-amino-2-chloropyrimidine-5-carboxylate (60 mg, 0.30 mmol) and the corresponding amine or the salt (0.45 mmol) was dissolved in 4 mL dry MeCN. Et_3N (91 mg, 0.90 mmol) was added to the solution. The mixture was stirred at room temperature for overnight. Removed the solvent and the crude product was purified by flash column chromatography on silica gel, eluting with hexanes/ethyl acetate (with 5% 7 N NH_3 in MeOH) (4:1 to 1:1) to afford the desired product as a white solid.

The product (0.20 mmol) above was dissolved in EtOH and H_2O (2 mL/2 mL). The mixture was heated to 60 °C for 4 h. After cooling to room temperature, EtOH was removed under reduced pressure. Saturated $NaHCO_3$ and 1 N HCl were used to make the solution to PH = 7. The solution was extracted with DCM (3×15 mL) and dried over Na_2SO_4. Removed the solvent and the residue was used for the next step directly. A solution of the crude product above, DIPEA (77 mg, 0.6 mmol) and HBTU (0.21 mmol) in DMF was stirred for 15 min. at room temperature. 4-methoxyaniline (26 mg, 0.21 mmol) in DMF (1 mL) was added to the solution. The mixture was stirred at room temperature overnight. The mixture was washed with 1 N NaOH solution and extracted with ethyl acetate (3×15 mL) and dried over Na_2SO_4. Removed the solvent and the residue was purified by flash column chromatography on silica gel, eluting with hexanes/ethyl acetate (with 5% 7 N NH_3 in MeOH) or DCM/MeOH to afford the desired product as a solid.

Characterization of Ester Intermediates

ethyl 4-amino-2-(4-(quinolin-3-yl)piperazin-1-yl)pyrimidine-5-carboxylate (GT-5-79)

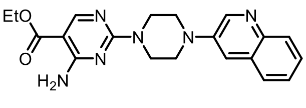

^{1}H NMR (400 MHz, Chloroform-*d*) δ 8.83 (d, *J* = 2.8 Hz, 1H), 8.68 (s, 1H), 8.14 (s, 1H), 7.84 (brsm, 1H), 7.76 – 7.69 (m, 1H), 7.61 – 7.47 (m, 3H), 5.46 (brsm, 1H), 4.31 (q, *J* = 7.1, 2H), 4.13 (s, 4H), 3.37 (d, *J* = 5.3 Hz, 4H), 1.36 (t, *J* = 7.1 Hz, 3H); **^{13}C NMR (101 MHz, Chloroform-*d*)** δ 166.4 , 163.2, 144.6, 142.6, 129.0, 127.9, 127.8, 127.6,127.4, 126.7, 96.6, 60.5, 49.0, 43.6, 14.4. **[M+H]$^+$LCMS**: 379; **HRMS** (ESI) calculated for C20H23N6O2$^+$ ([M+H$^+$]) 379.1877, found: 379.1911. White solid, 88 mg, 78%.

ethyl 4-amino-2-(4-(quinolin-2-yl)piperazin-1-yl)pyrimidine-5-carboxylate (GT-5-80)

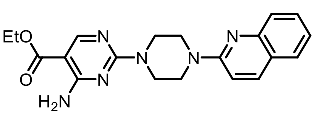

^{1}H NMR (400 MHz, Chloroform-*d*) δ 8.67 (s, 1H), 7.95 (d, *J* = 9.1 Hz, 1H), 7.74 (brsm, 1H), 7.65 – 7.52 (m, 2H), 7.26 (d, *J* = 3.0 Hz, 1H), 7.01 (d, *J* = 9.2 Hz, 1H), 5.29 (brsm, 1H), 4.29 (q, *J* = 7.1 Hz, 2H), 4.11 – 3.97 (m, 4H), 3.86 (s, 4H), 1.35 (t, *J* = 7.1 Hz, 3H); **^{13}C NMR (101 MHz, Chloroform-*d*)** δ 166.9, 163.4, 161.9, 161.0, 130.1, 127.4, 123.1, 109.8, 96.4, 60.3, 45.5, 43.6, 14.5. **[M+H]$^+$LCMS**: 379; **HRMS** (ESI) calculated for C20H23N6O2$^+$ ([M+H$^+$]) 379.1877, found: 379.1905. White solid, 93 mg, 82%.

ethyl 4-amino-2-(4-(isoquinolin-3-yl)piperazin-1-yl)pyrimidine-5-carboxylate (GT-5-91)

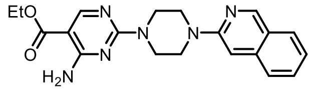

^{1}H NMR (400 MHz, Chloroform-*d*) δ 8.97 (s, 1H), 8.69 (s, 1H), 7.85 – 7.76 (m, 1H), 7.55 – 7.51 (m, 1H), 7.32 – 7.28 (m, 1H), 6.82 (s, 1H), 4.31 (q, *J* = 7.1 Hz, 2H), 4.10 (s, 4H), 3.68 (t, *J* = 5.3 Hz, 4H), 1.36 (t, *J* = 7.1 Hz, 4H); **^{13}C NMR (101 MHz, Chloroform-*d*)** δ 163.0, 156.1, 151.1, 149.0, 138.8, 131.3, 130.6, 127.7, 125.4, 123.8_5, 123.7_9, 122.8, 99.6, 60.7, 46.2, 44.0, 14.3. **[M+H]$^+$LCMS**: 379; **HRMS** (ESI) calculated for C20H23N6O2$^+$ ([M+H$^+$]) 379.1877, found: 379.1911. White solid, 91 mg, 80%.

ethyl 4-amino-2-(4-(benzo[d]oxazol-2-yl)piperazin-1-yl)pyrimidine-5-carboxylate (GT-5-100)

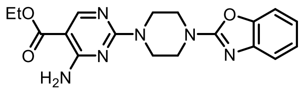

^{1}H NMR (400 MHz, $CDCl_3$) δ 8.67 (s, 1H), 7.76 (brsm, 1H), 7.38 (dd, *J* = 7.8, 1.2 Hz, 1H), 7.27 (dd, *J* = 9.3, 1.8 Hz, 1H), 7.18 (td, *J* = 7.7, 1.1 Hz, 1H), 7.04 (td, *J* = 7.7, 1.2 Hz, 1H), 5.33 (brsm, 1H), 4.30 (q, *J* = 7.1 Hz, 2H), 4.02 (t, *J* = 5.2 Hz, 4H), 3.80 – 3.70 (m, 4H), 1.35 (t, *J* = 7.1 Hz, 3H); **^{13}C NMR (101 MHz, $CDCl_3$)** δ 166.7, 163.4, 162.2, 148.9, 143.1, 124.3, 121.1, 116.6, 109.0, 96.7, 60.4, 45.6, 43.3, 14.5. **[M+H]$^+$LCMS**: 369; **HRMS** (ESI) calculated for C18H21N6O3$^+$ ([M+H$^+$]) 369.1670, found: 369.1678. White solid, 84 mg, 77%.

ethyl 4-amino-2-(4-(benzo[d]thiazol-2-yl)piperazin-1-yl)pyrimidine-5-carboxylate (GT-5-104)

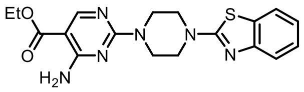

^{1}H NMR (400 MHz, DMSO) δ 8.54 (s, 1H), 7.78 (dd, *J* = 8.0, 1.2 Hz, 1H), 7.56 – 7.44 (m, 3H), 7.29 (td, *J* = 8.2, 1.3 Hz, 1H), 7.08 (td, *J* = 7.6, 1.2 Hz, 1H), 4.22 (q, *J* = 7.1 Hz, 2H), 3.95 (t, *J* = 5.2 Hz, 4H), 3.65 (t, *J* = 5.2 Hz, 4H), 1.28 (t, *J* = 7.1 Hz, 3H); **^{13}C NMR (101 MHz, DMSO)** δ 168.1, 165.9, 162.8, 161.3, 160.5, 152.3, 130.4, 126.0, 121.3, 121.2, 118.6, 95.2, 59.7, 47.7, 42.5, 14.2. **[M+H]$^+$LCMS**: 385; **HRMS** (ESI) calculated for C18H21N6O2S$^+$ ([M+H$^+$]) 385.1441, found: 385.1452. White solid, 86 mg, 76%.

ethyl 4-amino-2-(4-(4-methoxyphenyl)piperazin-1-yl)pyrimidine-5-carboxylate (GT-5-111)

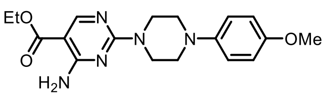

^{1}H NMR (400 MHz, DMSO) δ 8.52 (s, 1H), 7.43 (d, *J* = 28.6 Hz, 2H), 7.00 – 6.86 (m, 4H), 4.22 (q, *J* = 7.1 Hz, 2H), 3.91 (t, *J* = 5.0 Hz, 4H), 3.80 (s, 3H), 2.97 (t, *J* = 5.0 Hz, 4H), 1.27 (t, *J* = 7.1 Hz, 3H); **^{13}C NMR (101 MHz, DMSO)** δ 165.9, 162.8, 161.3, 160.4, 152.1, 141.0, 122.8, 120.8, 118.4, 111.9, 94.8, 59.6, 55.4, 50.1, 43.4, 14.2. **[M+H]$^+$LCMS**: 358; **HRMS** (ESI) calculated for C18H24N5O3$^+$ ([M+H$^+$]) 358.1874, found: 358.1877. White solid, 87 mg, 82%.

ethyl 4-amino-2-(4-(quinoxalin-2-yl)piperazin-1-yl)pyrimidine-5-carboxylate (GT-5-112)

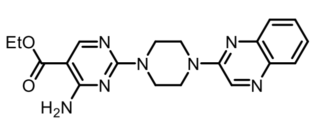

^{1}H NMR (400 MHz, DMSO) δ 8.75 (d, *J* = 1.8 Hz, 1H), 8.63 (d, *J* = 1.9 Hz, 1H), 8.55 (s, 1H), 7.91 (d, *J* = 9.3 Hz, 1H), 7.76 (dd, *J* = 9.4, 2.7 Hz, 1H), 7.48 (d, *J* = 7.5 Hz, 2H), 7.28 (d, *J* = 2.7 Hz, 1H), 4.22 (q, *J* = 7.1 Hz, 2H), 3.98 (t, *J* = 5.0 Hz, 4H), 3.47 (t, *J* = 5.2 Hz, 4H), 1.28 (t, *J* = 7.0 Hz, 3H); **^{13}C NMR (101 MHz, DMSO)** δ 165.9, 162.8, 161.3, 160.5, 151.4, 145.5, 144.1, 141.8, 137.4, 129.4, 122.3, 108.5, 95.0, 59.7, 47.4, 42.8, 14.2. **[M+H]$^+$LCMS**: 380; **HRMS** (ESI) calculated for C19H22N7O2$^+$ ([M+H$^+$]) 380.1829, found: 380.1861. Yellow solid, 90 mg, 80%.

ethyl 4-amino-2-(4-(pyridin-4-yl)piperazin-1-yl)pyrimidine-5-carboxylate (GT-9-38-1)

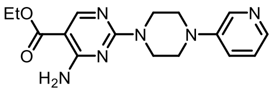

^{1}H NMR (400 MHz, DMSO) δ 8.53 (s, 1H), 8.34 (d, *J* = 2.9 Hz, 1H), 8.02 (dd, *J* = 4.5, 1.3 Hz, 1H), 7.46 (d, *J* = 13.0 Hz, 2H), 7.36 (ddd, *J* = 8.5, 3.0, 1.4 Hz, 1H), 7.23 (dd, *J* = 8.5, 4.5 Hz, 1H), 4.22 (q, *J* = 7.1 Hz, 2H), 3.93 (t, *J* = 5.1 Hz, 4H), 3.25 (t, *J* = 5.2 Hz, 4H), 1.27 (t, *J* = 7.1 Hz, 3H); **^{13}C NMR (101 MHz, DMSO)** δ 165.9, 162.8, 161.3, 160.5, 146.6, 140.0, 138.1, 123.5, 122.1, 95.0, 59.7, 47.5, 42.8, 14.2. **LCMS**: 329 [M+H]$^+$; **HRMS** (ESI) calculated for $C_{16}H_{21}N_6O_2^+$ ([M+H$^+$]) 329.1721, found: 329.1726. White solid, 55 mg, 56%.

ethyl 4-amino-2-(4-(pyridin-3-yl)piperazin-1-yl)pyrimidine-5-carboxylate (GT-9-39-1)

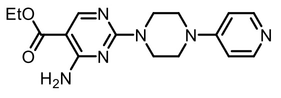

^{1}H NMR (400 MHz, DMSO) δ 8.53 (s, 1H), 8.18 (d, *J* = 6.5 Hz, 2H), 7.47 (d, *J* = 11.7 Hz, 2H), 6.84 (d, *J* = 6.7 Hz, 2H), 4.22 (q, *J* = 7.1 Hz, 2H), 3.89 (t, *J* = 5.3 Hz, 4H), 3.43 – 3.38 (m, 4H), 1.27 (t, *J* = 7.1 Hz, 3H); **^{13}C NMR (101 MHz, DMSO)** δ 165.9, 162.8, 161.3, 160.5, 154.3, 149.8, 108.3, 95.1, 59.7, 45.1, 42.6, 14.2. **LCMS**: 329 [M+H]$^+$; **HRMS** (ESI) calculated for $C_{16}H_{21}N_6O_2^+$ ([M+H$^+$]) 329.1721, found: 329.1709. White solid, 61 mg, 62%.

ethyl 4-amino-2-(4-(pyrimidin-2-yl)piperazin-1-yl)pyrimidine-5-carboxylate (GT-9-40-1)

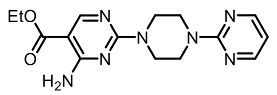

^{1}H NMR (400 MHz, DMSO) δ 8.53 (s, 1H), 8.38 (d, *J* = 4.7 Hz, 2H), 7.46 (d, *J* = 9.5 Hz, 2H), 6.65 (t, *J* = 4.7 Hz, 1H), 4.22 (q, *J* = 7.1 Hz, 2H), 3.86 (dd, *J* = 6.9, 3.8 Hz, 4H), 3.79 (dd, *J* = 7.1, 3.9 Hz, 4H), 1.27 (t, *J* = 7.1 Hz, 3H); **^{13}C NMR (101 MHz, DMSO)** δ 165.9, 162.8, 161.4, 161.1, 160.4, 157.9, 110.3, 95.0, 59.7, 43.1, 43.0, 14.2. **LCMS**: 330 [M+H]$^+$; **HRMS** (ESI) calculated for $C_{15}H_{20}N_7O_2^+$ ([M+H$^+$]) 330.1673, found: 330.1667. White solid, 67 mg, 68%.

ethyl 4-amino-2-(4-(pyrazin-2-yl)piperazin-1-yl)pyrimidine-5-carboxylate (GT-9-41-1)

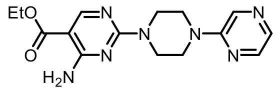

^{1}H NMR (400 MHz, DMSO) δ 8.53 (s, 1H), 8.36 (d, *J* = 1.6 Hz, 1H), 8.10 (dd, *J* = 2.6, 1.5 Hz, 1H), 7.86 (d, *J* = 2.6 Hz, 1H), 7.47 (d, *J* = 7.2 Hz, 2H), 4.22 (q, *J* = 7.1 Hz, 2H), 3.91 – 3.88 (m, 4H), 3.67 – 3.62 (m, 4H), 1.27 (t, *J* = 7.1 Hz, 3H); **^{13}C NMR (101 MHz, DMSO)** δ 165.9, 162.8, 161.3, 160.5, 154.5, 141.4, 132.6, 131.4, 95.0, 59.7, 43.7, 42.7, 14.2. **LCMS**: 330 [M+H]$^+$; **HRMS** (ESI) calculated for $C_{15}H_{20}N_7O_2^+$ ([M+H$^+$]) 330.1673, found: 330.1693. White solid, 70 mg, 71%.

ethyl 4-amino-2-(4-(thiazol-2-yl)piperazin-1-yl)pyrimidine-5-carboxylate (GT-9-42-1)

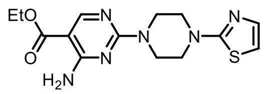

^{1}H NMR (400 MHz, DMSO) δ 8.53 (s, 1H), 7.47 (d, *J* = 5.8 Hz, 2H), 7.18 (d, *J* = 3.6 Hz, 1H), 6.87 (d, *J* = 3.6 Hz, 1H), 4.22 (q, *J* = 7.1 Hz, 2H), 3.91 (t, *J* = 5.2 Hz, 4H), 3.50 – 3.43 (m, 4H), 1.27 (t, *J* = 7.1 Hz, 3H).; **^{13}C NMR (101 MHz, DMSO)** δ 171.3, 165.9, 162.8, 161.3, 160.5, 139.4, 108.3, 95.2, 59.7, 48.0, 42.4, 14.2. **LCMS**: 335 [M+H]$^+$; **HRMS** (ESI) calculated for $C_{14}H_{19}N_6O_2S^+$ ([M+H$^+$]) 335.1285, found: 335.1304. White solid, 57 mg, 57%.

ethyl 4-amino-2-(4-(6-fluoropyridin-2-yl)piperazin-1-yl)pyrimidine-5-carboxylate (GT-9-63-1)

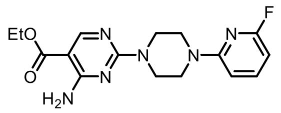

^{1}H NMR (400 MHz, DMSO) δ 8.53 (s, 1H), 7.75 – 7.64 (m, 1H), 7.46 (d, *J* = 6.9 Hz, 2H), 6.72 (dd, *J* = 8.3, 2.6 Hz, 1H), 6.29 (dd, *J* = 7.7, 2.7 Hz, 1H), 4.22 (q, *J* = 7.1 Hz, 2H), 3.87 (t, *J* = 5.3 Hz, 4H), 3.61 – 3.52 (m, 4H), 1.27 (t, *J* = 7.1 Hz, 3H); **^{13}C NMR (101 MHz, DMSO)** δ 165.9, 162.8, 162.2 (d, *J* = 233.5 Hz), 161.3, 160.4, 157.8 (d, *J* = 16.1 Hz), 142.7 (d, *J* = 8.3 Hz), 103.5 (d, *J* = 4.0 Hz), 95.4 (d, *J* = 36.9 Hz), 95.0, 59.7, 44.1, 42.7, 14.2. **LCMS**: 347 [M+H]$^+$; **HRMS** (ESI) calculated for $C_{16}H_{20}FN_6O_2^+$ ([M+H$^+$]) 347.1626, found: 347.1651. White solid, 71 mg, 69%.

ethyl 4-amino-2-(4-(5-fluoropyridin-2-yl)piperazin-1-yl)pyrimidine-5-carboxylate (GT-9-64-1)

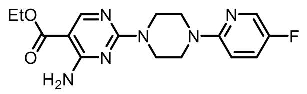

^{1}H NMR (400 MHz, DMSO) δ 8.53 (s, 1H), 8.11 (d, *J* = 3.1 Hz, 1H), 7.54 (ddd, *J* = 9.3, 8.3, 3.1 Hz, 1H), 7.45 (d, *J* = 11.2 Hz, 2H), 6.92 (dd, *J* = 9.3, 3.4 Hz, 1H), 4.22 (q, *J* = 7.1 Hz, 2H), 3.91 – 3.85 (m, 4H), 3.53 – 3.48 (m, 4H), 1.27 (t, *J* = 7.1 Hz, 3H); **^{13}C NMR (101 MHz, DMSO)** δ 165.9, 161.6 (d, *J* = 239.1 Hz), 161.3, 156.0, 154.3, 151.9, 134.0 (d, *J* = 23.9 Hz), 125.3 (d, *J* = 20.0 Hz), 108.3 (d, *J* = 3.9 Hz), 95.0, 59.7, 45.1, 42.8, 14.2. **LCMS**: 347 [M+H]$^+$; **HRMS** (ESI) calculated for $C_{16}H_{20}FN_6O_2^+$ ([M+H$^+$]) 347.1626, found: 347.1638. White solid, 68 mg, 66%.

ethyl 4-amino-2-(4-(4-fluoropyridin-2-yl)piperazin-1-yl)pyrimidine-5-carboxylate (GT-9-67-1)

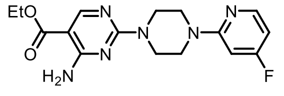

^{1}H NMR (400 MHz, DMSO) δ 8.53 (s, 1H), 8.13 (dd, *J* = 9.8, 5.7 Hz, 1H), 7.46 (d, *J* = 7.5 Hz, 2H), 6.72 (dd, *J* = 13.3, 2.2 Hz, 1H), 6.55 (ddd, *J* = 8.0, 5.7, 2.1 Hz, 1H), 4.22 (q, *J* = 7.1 Hz, 2H), 3.88 – 3.85 (m, 4H), 3.63 – 3.56 (m, 4H), 1.27 (t, *J* = 7.1 Hz, 3H); **^{13}C NMR (101 MHz, DMSO)** δ 169.8 (d, *J* = 253.6 Hz), 165.9, 162.8, 161.3, 161.0 (d, *J* = 10.7 Hz), 160.4, 150.2 (d, *J* = 9.5 Hz), 101.2 (d, *J* = 18.0 Hz), 95.0, 93.5 (d, *J* = 21.5 Hz), 59.7, 44.3, 42.8, 14.2. **LCMS**: 347 [M+H]$^+$; **HRMS** (ESI) calculated for $C_{16}H_{20}FN_6O_2^+$ ([M+H$^+$]) 347.1626, found: 347.1652. White solid, 76 mg, 74%.

ethyl 4-amino-2-(4-(3-fluoropyridin-2-yl)piperazin-1-yl)pyrimidine-5-carboxylate (GT-9-68-1)

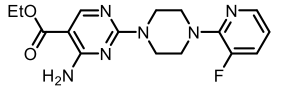

^{1}H NMR (400 MHz, DMSO) δ 8.53 (s, 1H), 8.03 (dt, *J* = 4.8, 1.6 Hz, 1H), 7.54 (ddd, *J* = 13.7, 7.9, 1.5 Hz, 1H), 7.45 (d, *J* = 15.5 Hz, 2H), 6.91 (ddd, *J* = 7.9, 4.8, 3.1 Hz, 1H), 4.22 (q, *J* = 7.1 Hz, 2H), 3.96 – 3.86 (m, 4H), 3.47 – 3.40 (m, 4H), 1.27 (t, *J* = 7.1 Hz, 3H); **^{13}C NMR (101 MHz, DMSO)** δ 165.9, 161.6 (d, *J* = 240.3 Hz), 161.3, 150.7, 149.2 (d, *J* = 6.4 Hz), 148.1, 142.9 (d, *J* = 5.4 Hz), 123.7 (d, *J* = 18.6 Hz), 116.5, 95.0, 59.7, 47.1, 43.0, 14.2. **LCMS**: 347 [M+H]$^+$; **HRMS** (ESI) calculated for $C_{16}H_{20}FN_6O_2^+$ ([M+H$^+$]) 347.1626, found: 347.1653. White solid, 72 mg, 70%.

ethyl 4-amino-2-(4-(pyridin-2-yl)piperazin-1-yl)pyrimidine-5-carboxylate (GT-5-46)

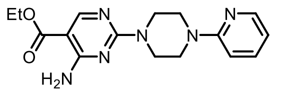

^{1}H NMR (400 MHz, DMSO) δ 8.52 (s, 1H), 8.12 (ddd, *J* = 4.9, 2.0, 0.8 Hz, 1H), 7.55 (ddd, *J* = 8.9, 7.1, 2.0 Hz, 1H), 7.47 (s, 1H), 7.41 (s, 1H), 6.86 (d, *J* = 8.6 Hz, 1H), 6.66 (dd, *J* = 6.7, 4.5 Hz, 1H), 4.21 (q, *J* = 7.1 Hz, 2H), 1.26 (t, *J* = 7.1 Hz, 3H); **^{13}C NMR (101 MHz, DMSO)** δ 166.1, 162.9, 161.5, 160.6, 158.9, 147.7, 137.8, 113.4, 107.4, 95.1, 59.9, 44.5, 43.0, 14.4. **[M+H]$^+$LCMS**: 329; **HRMS** (ESI) calculated for $C_{16}H_{21}N_6O_2^+$ ([M+H$^+$]) 329.1721, found: 329.1745. White solid, 85 mg, 51%.

Characterization of Final Products

4-amino-*N*-(4-methoxyphenyl)-2-(4-(quinolin-3-yl)piperazin-1-yl)pyrimidine-5-carboxamide (GT-5-83)

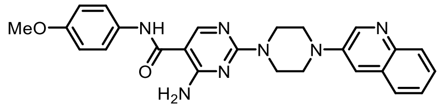

^{1}H NMR (400 MHz, DMSO-*d*$_6$) δ 9.77 (s, 1H), 8.91 (d, *J* = 2.8 Hz, 1H), 8.62 (s, 1H), 7.91 – 7.85 (m, 1H), 7.82 – 7.76 (m, 1H), 7.59 (d, *J* = 2.9 Hz, 1H), 7.53 (d, *J* = 9.0 Hz, 2H), 7.51 – 7.48 (m, 2H), 6.89 (d, *J* = 9.0 Hz, 2H), 3.98 (t, *J* = 5.0 Hz, 4H), 3.36 (t, *J* = 5.0 Hz, 4H); **^{13}C NMR (101 MHz, DMSO-*d*$_6$)** δ 165.8 , 163.4 , 162.9, 161.3 , 157.9 , 155.9 , 145.1 , 145.0 , 142.6 , 132.4 , 129.1 , 128.8 , 127.3 , 127.2 , 126.6 , 122.8 , 118.6 , 116.5 , 114.2 , 99.6, 55.6, 48.7, 43.4. **[M+H]$^+$LCMS**: 456; **HRMS** (ESI) calculated for C25H26N7O2$^+$ ([M+H$^+$] 456.2142, found: 456.2152. Brown solid, 51 mg, 56% for 2 steps.

4-amino-*N*-(4-methoxyphenyl)-2-(4-(quinolin-2-yl)piperazin-1-yl)pyrimidine-5-carboxamide (GT-5-84)

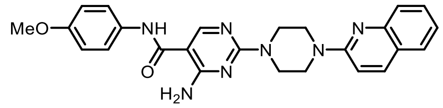

^{1}H NMR (400 MHz, DMSO-*d*$_6$) δ 9.75 (s, 1H), 8.63 (s, 1H), 8.08 (d, *J* = 9.1 Hz, 1H), 7.72 (dd, *J* = 8.2, 1.4 Hz, 1H), 7.61 – 7.57 (m, 1H), 7.56 – 7.51 (m, 3H), 7.30 (d, *J* = 9.0 Hz, 1H), 7.24 (ddd, *J* = 8.1, 6.7, 1.3 Hz, 1H), 6.90 (d, *J* = 9.0 Hz, 2H), 3.92 (dd, *J* = 6.9, 3.7 Hz, 4H), 3.78 (dd, *J* = 6.8, 3.9 Hz, 4H), 3.73 (s, 3H); **^{13}C NMR (101 MHz, DMSO-*d*$_6$)** δ 165.8, 163.4, 161.4, 157.9, 157.5, 155.9, 147.7, 137.9, 132.5, 129.9, 127.9, 126.5, 123.3, 122.7, 122.6, 114.1, 110.7, 99.5, 55.6, 44.9, 43.5. **[M+H]$^+$LCMS**: 456; **HRMS** (ESI) calculated for C25H26N7O2$^+$ ([M+H$^+$] 456.2142, found: 456.2165. Yellow solid, 46 mg, 50% for 2 steps.

4-amino-2-(4-(isoquinolin-3-yl)piperazin-1-yl)-*N*-(4-methoxyphenyl)pyrimidine-5-carboxamide (GT-5-93)

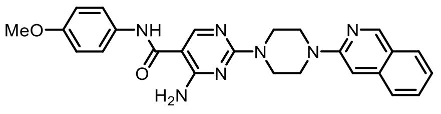

^{1}H NMR (400 MHz, DMSO-*d*$_6$) δ 9.76 (s, 1H), 9.01 (s, 1H), 8.63 (s, 1H), 7.89 (d, *J* = 8.2 Hz, 1H), 7.69 (d, *J* = 8.4 Hz, 1H), 7.58 – 7.52 (m, 3H), 7.29 (t, *J* = 7.5 Hz, 1H), 7.04 (s, 1H), 6.90 (d, *J* = 9.0 Hz, 2H), 3.94 (t, *J* = 5.2 Hz, 4H), 3.73 (s, 3H), 3.63 (t, *J* = 5.2 Hz, 4H); **^{13}C NMR (101 MHz, DMSO-*d*$_6$)** δ 165.8, 163.4, 161.4, 157.9, 156.5, 155.9, 151.4, 138.8, 132.5, 130.9, 128.0, 125.6, 123.7, 123.5, 122.8, 114.1, 99.5, 99.0, 55.6, 45.8, 43.4. **[M+H]$^+$LCMS**: 456; **HRMS** (ESI) calculated for C25H26N7O2$^+$ ([M+H$^+$] 456.2142, found: 456.2133. Yellow solid, 44 mg, 48% for 2 steps.

4-amino-2-(4-(benzo[*d*]oxazol-2-yl)piperazin-1-yl)-*N*-(4-methoxyphenyl)pyrimidine-5-carboxamide (GT-5-105)

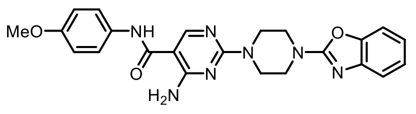

^{1}H NMR (400 MHz, DMSO) δ 9.77 (s, 1H), 8.62 (s, 1H), 7.54 (d, *J* = 9.1 Hz, 2H), 7.42 (d, *J* = 7.7 Hz, 1H), 7.31 (d, *J* = 7.7 Hz, 1H), 7.17 (td, *J* = 7.7, 1.1 Hz, 1H), 7.04 (td, *J* = 7.7, 1.1 Hz, 1H), 6.90 (d, *J* = 9.1 Hz, 2H), 3.94 (t, *J* = 5.2 Hz, 4H), 3.73 (s, 3H), 3.68 (t, *J* = 5.2 Hz, 4H); **^{13}C NMR (101 MHz, DMSO)** δ 165.3, 162.9, 161.8, 160.8, 157.4, 155.4, 148.3, 142.9, 132.0, 124.0, 122.3, 120.6, 115.9, 113.7, 108.9, 99.3, 55.2, 45.1, 42.5. **[M+H]$^+$LCMS**: 446; **HRMS** (ESI) calculated for C23H24N7O3$^+$ ([M+H$^+$]) 446.1935, found: 446.1941. White solid, 39 mg, 44% for 2 steps.

4-amino-2-(4-(benzo[d]thiazol-2-yl)piperazin-1-yl)-*N*-(4-methoxyphenyl)pyrimidine-5-carboxamide (GT-5-107)

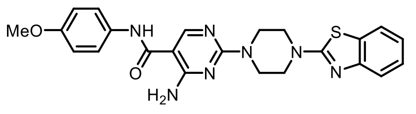

^{1}H NMR (400 MHz, DMSO) δ 9.77 (d, *J* = 3.9 Hz, 1H), 8.62 (d, *J* = 4.4 Hz, 1H), 7.79 (s, 1H), 7.56 – 7.46 (m, 3H), 7.29 (d, *J* = 6.9 Hz, 1H), 7.09 (d, *J* = 7.0 Hz, 1H), 6.90 (s, 2H), 3.94 (s, 4H), 3.73 (s, 3H), 3.66 (s, 4H); **^{13}C NMR (101 MHz, DMSO)** δ 168.2, 165.3, 162.9, 160.8, 157.5, 155.4, 152.4, 132.0, 130.4, 126.0, 122.3, 121.4, 121.2, 118.7, 113.7, 99.3, 55.2, 47.8, 42.6. **[M+H]$^+$LCMS**: 462; **HRMS** (ESI) calculated for C23H24N7O2S$^+$ ([M+H$^+$]) 462.1707, found: 4621705. White solid, 48 mg, 51% for 2 steps.

4-amino-*N*-(4-methoxyphenyl)-2-(4-(quinoxalin-2-yl)piperazin-1-yl)pyrimidine-5-carboxamide (GT-5-119)

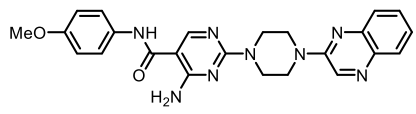

^{1}H NMR (400 MHz, DMSO) δ 9.76 (s, 1H), 8.76 (d, *J* = 1.9 Hz, 1H), 8.65 – 8.60 (m, 2H), 7.92 (d, *J* = 9.3 Hz, 1H), 7.78 (dd, *J* = 9.3, 2.7 Hz, 1H), 7.54 (d, *J* = 8.9 Hz, 2H), 7.29 (d, *J* = 2.7 Hz, 1H), 6.90 (d, *J* = 8.9 Hz, 2H), 3.97 (t, *J* = 5.2 Hz, 4H), 3.73 (s, 3H), 3.48 (t, *J* = 5.2 Hz, 4H); **^{13}C NMR (101 MHz, DMSO)** δ 165.3, 162.9, 160.8, 157.5, 155.4, 151.5, 145.5, 144.1, 141.9, 137.4, 132.0, 129.5, 122.4, 122.3, 113.7, 108.6, 99.1, 55.2, 47.5, 42.8. **[M+H]$^+$LCMS**: 462; **HRMS** (ESI) calculated for C24H25N8O2$^+$ ([M+H$^+$])$^+$]) 457.2095, found: 457.2098. White solid, 48 mg, 51% for 2 steps.

4-amino-*N*-(4-methoxyphenyl)-2-(4-(4-methoxyphenyl)piperazin-1-yl)pyrimidine-5-carboxamide (GT-5-118)

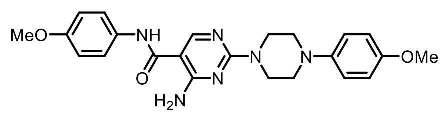

^{1}H NMR (400 MHz, DMSO) δ 9.74 (s, 1H), 8.61 (s, 1H), 7.55 (s, 3H), 6.96 – 6.88 (m, 5H), 3.90 (s, 4H), 3.81 (s, 3H), 3.73 (s, 3H), 2.98 (s, 4H); **^{13}C NMR (101 MHz, DMSO)** δ 165.4, 162.9, 160.8, 157.5, 155.4, 152.1, 141.1, 132.1, 122.8, 122.2, 120.8, 118.4, 113.7, 111.9, 98.9, 55.4, 55.2, 50.2, 43.4. **[M+H]$^+$LCMS**: 457; **HRMS** (ESI) calculated for C23H27N6O3$^+$ ([M+H$^+$]) 435.2139, found: 435.2151. White solid, 36 mg, 41% for 2 steps.

4-amino-*N*-(4-methoxyphenyl)-2-(4-(pyridin-3-yl)piperazin-1-yl)pyrimidine-5-carboxamide (GT-9-38)

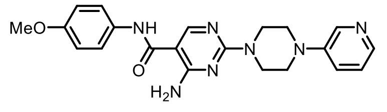

^{1}H NMR (400 MHz, DMSO) δ 9.74 (s, 1H), 8.62 (s, 1H), 8.35 (d, *J* = 2.9 Hz, 1H), 8.02 (d, *J* = 4.4 Hz, 1H), 7.54 (d, *J* = 9.1 Hz, 3H), 7.37 (ddd, *J* = 8.6, 3.0, 1.4 Hz, 1H), 7.23 (dd, *J* = 8.5, 4.5 Hz, 1H), 6.89 (d, *J* = 9.1 Hz, 2H), 3.92 (t, *J* = 5.2 Hz, 4H), 3.73 (s, 3H), 3.25 (t, *J* = 5.3 Hz, 4H); **^{13}C NMR (101 MHz, DMSO)** δ 165.3, 162.9, 160.8, 157.4, 155.4, 146.7, 140.0, 138.1, 132.0, 123.5, 122.2,

122.1, 113.7, 99.1, 55.2, 47.6, 42.8. **LCMS**: 406 $[M+H]^+$; **HRMS** (ESI) calculated for $C_{21}H_{24}N_7O_2^+$ ($[M+H^+]$) 406.1986, found: 406.1998. White solid, 32 mg, 39%.

4-amino-*N*-(4-methoxyphenyl)-2-(4-(pyridin-4-yl)piperazin-1-yl)pyrimidine-5-carboxamide (GT-9-39)

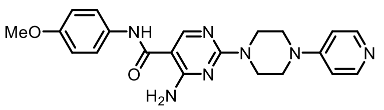

^{1}H NMR (400 MHz, DMSO) δ 9.75 (s, 1H), 8.62 (s, 1H), 8.25 – 8.14 (m, 2H), 7.61 – 7.48 (m, 3H), 6.89 (d, *J* = 9.0 Hz, 2H), 6.85 (d, *J* = 6.7 Hz, 2H), 3.89 (t, *J* = 5.3 Hz, 4H), 3.73 (s, 3H), 3.44 – 3.39 (m, 4H); **^{13}C NMR (101 MHz, DMSO)** δ 165.3, 162.9, 160.8, 157.4, 155.4, 154.4, 149.8, 132.0, 122.2, 113.7, 108.4, 99.1, 55.1, 45.2, 42.6. **LCMS**: 406 $[M+H]^+$; **HRMS** (ESI) calculated for $C_{21}H_{24}N_7O_2^+$ ($[M+H^+]$) 406.1986, found: 406.1988. White solid, 45 mg, 56%.

4-amino-*N*-(4-methoxyphenyl)-2-(4-(pyrimidin-2-yl)piperazin-1-yl)pyrimidine-5-carboxamide (GT-9-40)

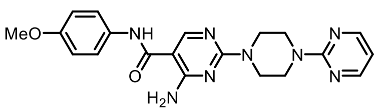

^{1}H NMR (400 MHz, DMSO) δ 9.74 (s, 1H), 8.61 (s, 1H), 8.39 (d, *J* = 4.8 Hz, 2H), 7.53 (d, *J* = 9.1 Hz, 3H), 6.89 (d, *J* = 9.0 Hz, 2H), 6.66 (t, *J* = 4.7 Hz, 1H), 3.85 (d, *J* = 4.9 Hz, 4H), 3.82 – 3.77 (m, 4H), 3.73 (s, 3H); **^{13}C NMR (101 MHz, DMSO)** δ 165.8, 163.4, 161.7, 161.4, 158.5, 157.9, 155.9, 132.5, 122.8, 114.1, 110.8, 99.5, 55.6, 43.6, 43.4. **LCMS**: 407 $[M+H]^+$; **HRMS** (ESI) calculated for $C_{20}H_{23}N_8O_2^+$ ($[M+H^+]$) 407.1938, found: 407.1968. White solid, 47 mg, 58%.

4-amino-*N*-(4-methoxyphenyl)-2-(4-(pyrazin-2-yl)piperazin-1-yl)pyrimidine-5-carboxamide (GT-9-41)

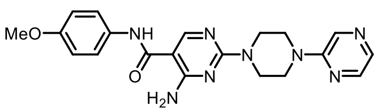

^{1}H NMR (400 MHz, DMSO) δ 9.75 (s, 1H), 8.62 (s, 1H), 8.37 (d, *J* = 1.6 Hz, 1H), 8.11 (dd, *J* = 2.6, 1.5 Hz, 1H), 7.86 (d, *J* = 2.6 Hz, 1H), 7.54 (d, *J* = 9.1 Hz, 3H), 6.89 (d, *J* = 9.1 Hz, 2H), 3.93 – 3.86 (m, 4H), 3.73 (s, 3H), 3.68 – 3.62 (m, 4H); **^{13}C NMR (101 MHz, DMSO)** δ 165.3, 162.9, 160.8, 157.4, 155.4, 154.6, 141.4, 132.6, 132.0, 131.4, 122.3, 113.7, 99.1, 55.1, 43.7, 42.7. **LCMS**: 407 $[M+H]^+$; **HRMS** (ESI) calculated for $C_{20}H_{23}N_8O_2^+$ ($[M+H^+]$) 407.1938, found: 407.1956. White solid, 45 mg, 55%.

4-amino-*N*-(4-methoxyphenyl)-2-(4-(thiazol-2-yl)piperazin-1-yl)pyrimidine-5-carboxamide (GT-9-42)

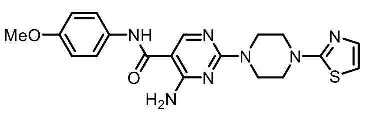

^{1}H NMR (400 MHz, DMSO) δ 9.75 (s, 1H), 8.61 (s, 1H), 7.53 (d, *J* = 9.0 Hz, 3H), 7.19 (d, *J* = 3.6 Hz, 1H), 6.93 – 6.85 (m, 3H), 3.91 (t, *J* = 5.3 Hz, 4H), 3.73 (s, 3H), 3.47 (t, *J* = 5.2 Hz, 4H); **^{13}C NMR (101 MHz, DMSO)** δ 171.4, 165.3, 162.9, 160.8, 157.4, 155.4, 139.4, 132.0, 122.3, 113.7, 108.3, 99.2, 55.2, 48.1, 42.4. **LCMS**: 412 $[M+H]^+$; **HRMS** (ESI) calculated for $C_{19}H_{22}N_7O_2S^+$ ($[M+H^+]$) 412.1550, found: 412.1566. White solid, 57 mg, 70%.

4-amino-2-(4-(6-fluoropyridin-2-yl)piperazin-1-yl)-*N*-(4-methoxyphenyl)pyrimidine-5-carboxamide (GT-9-63)

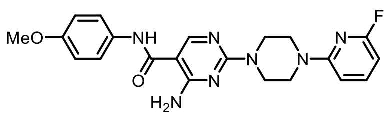

^{1}H NMR (400 MHz, DMSO) δ 9.75 (s, 1H), 8.62 (s, 1H), 7.69 (dt, *J* = 9.1, 7.9 Hz, 1H), 7.62 – 7.47 (m, 3H), 6.89 (d, *J* = 9.1 Hz, 2H), 6.73 (dd, *J* = 8.3, 2.6 Hz, 1H), 6.29 (dd, *J* = 7.7, 2.7 Hz, 1H), 3.92 – 3.83 (m, 4H), 3.73 (s, 3H), 3.61 – 3.53 (m, 4H); **^{13}C NMR (101 MHz, DMSO)** δ 165.3, 162.9, 162.0 (d, *J* = 233.5 Hz), 160.8, 157.9 (d, *J* = 15.8 Hz), 157.4, 155.4, 142.6 (d, *J* = 8.5 Hz), 132.0, 122.3, 113.7, 103.5 (d, *J* = 3.9 Hz), 99.1, 95.4 (d, *J* = 37.0 Hz), 55.1, 44.2, 42.7. **LCMS**: 424 $[M+H]^+$; **HRMS** (ESI) calculated for $C_{21}H_{23}FN_7O_2^+$ ($[M+H^+]$) 424.1892, found: 424.1915. White solid, 43 mg, 51%.

4-amino-2-(4-(5-fluoropyridin-2-yl)piperazin-1-yl)-*N*-(4-methoxyphenyl)pyrimidine-5-carboxamide (GT-9-64)

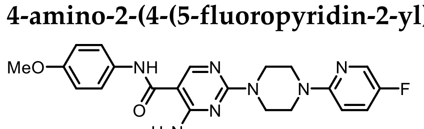

^{1}H NMR (400 MHz, DMSO) δ 9.74 (s, 1H), 8.61 (s, 1H), 8.12 (d, *J* = 3.1 Hz, 1H), 7.59 – 7.49 (m, 4H), 6.96 – 6.87 (m, 3H), 3.91 – 3.84 (m, 4H), 3.73 (s, 3H), 3.55 – 3.47 (m, 4H); **^{13}C NMR (101 MHz, DMSO)** δ 164.1 (d, *J* = 245.4 Hz), 160.8, 157.4, 156.1, 155.4,

151.9, 134.0 (d, *J* = 23.9 Hz), 132.0, 125.3 (d, *J* = 20.2 Hz), 122.3, 113.7, 108.3 (d, *J* = 3.7 Hz), 99.1, 55.1, 45.1, 42.8. **LCMS**: 424 $[M+H]^+$; **HRMS** (ESI) calculated for $C_{21}H_{23}FN_7O_2^+$ ($[M+H^+]$) 424.1892, found: 424.1920. White solid, 28 mg, 33%.

4-amino-2-(4-(4-fluoropyridin-2-yl)piperazin-1-yl)-*N*-(4-methoxyphenyl)pyrimidine-5-carboxamide (GT-9-67)

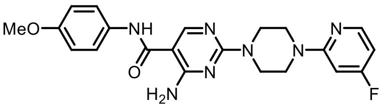

^{1}H NMR (400 MHz, DMSO) δ 9.74 (s, 1H), 8.61 (s, 1H), 8.14 (dd, *J* = 9.9, 5.7 Hz, 1H), 7.66 – 7.46 (m, 3H), 6.89 (d, *J* = 9.1 Hz, 2H), 6.73 (dd, *J* = 13.3, 2.2 Hz, 1H), 6.55 (ddd, *J* = 8.0, 5.7, 2.1 Hz, 1H), 3.90 – 3.81 (m, 4H), 3.73 (s, 3H), 3.64 – 3.56 (m, 4H); **^{13}C NMR (101 MHz, DMSO)** δ 164.1 (d, *J* = 243.9 Hz), 161.2, 161.1, 155.3 (d, *J* = 21.0 Hz), 150.3 (d, *J* = 8.9 Hz), 135.0, 132.0, 122.3, 113.7, 101.2 (d, *J* = 17.6 Hz), 99.1, 93.5 (d, *J* = 21.5 Hz), 55.2, 44.4, 42.7. **LCMS**: 424 $[M+H]^+$; **HRMS** (ESI) calculated for $C_{21}H_{23}FN_7O_2^+$ ($[M+H^+]$) 424.1892, found: 424.1902. White solid, 48 mg, 57%.

4-amino-2-(4-(3-fluoropyridin-2-yl)piperazin-1-yl)-*N*-(4-methoxyphenyl)pyrimidine-5-carboxamide (GT-9-68)

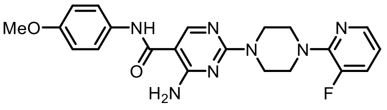

^{1}H NMR (400 MHz, DMSO) δ 9.74 (s, 1H), 8.61 (s, 1H), 8.04 (dt, *J* = 4.8, 1.5 Hz, 1H), 7.60 – 7.49 (m, 4H), 6.94 – 6.85 (m, 3H), 3.94 – 3.87 (m, 4H), 3.73 (s, 3H), 3.48 – 3.40 (m, 4H); ^{13}C NMR (101 MHz, DMSO) δ 164.1 (d, *J* = 246.3 Hz), 160.9, 157.4, 155.4, 150.7, 149.3 (d, *J* = 6.3 Hz), 148.2, 142.9 (d, *J* = 5.4 Hz), 132.0, 123.7 (d, *J* = 18.6 Hz), 122.3, 116.5, 113.7, 99.1, 55.2, 47.2 (d, *J* = 5.3 Hz), 43.0. LCMS: 424 $[M+H]^+$; HRMS (ESI) calculated for $C_{21}H_{23}FN_7O_2^+$ ($[M+H^+]$) 424.1892, found: 424.1902. White solid, 39 mg, 46%.

Methods for the synthesis of GT-9-36, GT-9-37 and related data.

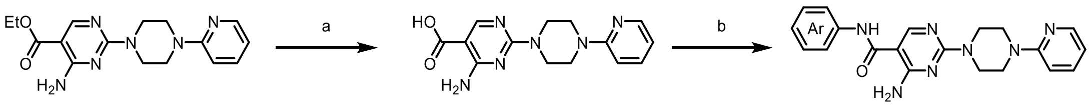

Step a: **GT-5-46** (0.20 mmol) was dissolved in EtOH and H_2O (2 mL/2 mL). The mixture was heated to 60 °C for 4 h. After cooling to room temperature, EtOH was removed under reduced pressure. 1 N HCl was used to make the solution to PH = 7. The precipitated solid was filtered out and dried on a high vacuum for 12 h. The crude product was used for the next step directly.

Step b: A solution of the crude product above, DIPEA (77 mg, 0.6 mmol) and HBTU (0.21 mmol) in DMF (4 mL) was stirred for 15 min. at room temperature. Corresponding amine (0.21 mmol) in DMF (1 mL) was added to the solution. The mixture was heated to 50 °C and stirred overnight. After cooling to room temperature, the mixture was washed with 1 N Na_2CO_3 solution and extracted with DCM (3× 15 mL), and dried over Na_2SO_4. Removed the solvent and the residue was purified by flash column chromatography on silica gel, eluting with 5% 7 N NH_3 MeOH in DCM to afford the desired product as a solid.

4-amino-*N*-(2-fluoro-4-methoxyphenyl)-2-(4-(pyridin-2-yl)piperazin-1-yl)pyrimidine-5-carboxamide (GT-9-36)

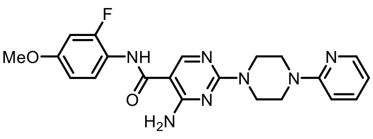

^{1}H NMR (400 MHz, DMSO) δ 9.58 (s, 1H), 8.65 (s, 1H), 8.13 (dd, *J* = 5.3, 1.9 Hz, 1H), 7.58 – 7.54 (m, 3H), 7.32 (t, *J* = 8.9 Hz, 1H), 6.96 – 6.83 (m, 2H), 6.77 (dd, *J* = 8.9, 2.3 Hz, 1H), 6.66 (dd, *J* = 7.1, 4.9 Hz, 1H), 3.87 (t, *J* = 5.3 Hz, 4H), 3.77 (s, 3H), 3.56 (t, *J* = 5.3 Hz, 4H); **^{13}C NMR (101 MHz, DMSO)** δ 165.8, 162.9, 160.9, 158.9, 158.2 (d, *J* = 228.4 Hz), 157.8, 156.9 (d, *J* = 7.3 Hz), 147.6, 137.6, 128.6 (d, *J* = 3.4 Hz), 118.0 (d, *J* = 13.0 Hz), 113.2, 109.7 (d, *J* = 3.1 Hz), 107.2, 101.9 (d, *J* = 23.9 Hz), 98.2, 55.7, 44.4, 42.9. **LCMS**: 424 $[M+H]^+$; **HRMS** (ESI) calculated for $C_{21}H_{23}FN_7O_2^+$ ($[M+H^+]$) 424.1892, found: 424.1887. White solid, 42 mg, 50%.

4-amino-*N*-(3-fluoro-4-methoxyphenyl)-2-(4-(pyridin-2-yl)piperazin-1-yl)pyrimidine-5-carboxamide (GT-9-37)

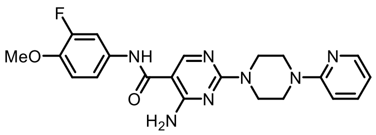

^{1}H NMR (400 MHz, DMSO) δ 9.86 (s, 1H), 8.61 (s, 1H), 8.13 (dd, *J* = 4.9, 1.7 Hz, 1H), 7.70 – 7.47 (m, 4H), 7.36 (ddd, *J* = 9.0, 2.6, 1.4 Hz, 1H), 7.12 (t, *J* = 9.3 Hz, 1H), 6.87 (d, *J* = 8.6 Hz, 1H), 6.66 (dd, *J* = 7.1, 4.9 Hz, 1H), 3.90 – 3.84 (m, 4H), 3.81 (s, 3H), 3.60 – 3.54 (m, 4H); **^{13}C NMR (101 MHz, DMSO)** δ 165.5, 162.8, 160.9, 158.9, 159.9, 157.6, 154.9 (d, *J* = 3.0 Hz), 151.9, 147.5, 137.6, 116.4 (d, *J* = 3.0 Hz), 113.8, 113.2, 109.0, 107.2, 98.8, 56.2, 44.4, 42.9.**LCMS**: 424 $[M+H]^+$; **HRMS** (ESI) calculated for $C_{21}H_{23}FN_7O_2^+$ ($[M+H^+]$) 424.1892, found: 424.1906. White solid, 45 mg, 53%.

Methods for the synthesis of GT-5-121 and related data.

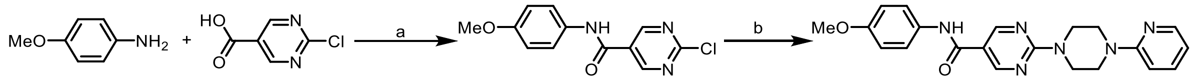

Conditions: a. $SOCl_2$, DCM, 0 °C to rt., overnight; b. 1-(pyridin-2-yl)piperazine, MeCN,Et_3N, rt. overnight.

Step a: A solution of ethyl 2-chloropyrimidine-5-carboxylic acid (317 mg, 2.0 mmol) in DCM (6 mL) was cooled to 0 °C under N_2. $SOCl_2$ (357 mg, 3.0 mmol) was added to the solution. The mixture was stirred for 3 h at room temperature. The extra $SOCl_2$ was removed under vacuum. The residue was dissolved in 6 mL DCM. 4-methoxyaniline (246 mg, 2.0 mmol) was added to the mixture. The mixture was stirred at room temperature overnight. 10 mL saturated $NaHCO_3$ was added to quench the reaction. The mixture was extracted with DCM 3 times. Combine the organic phase and dried with Na_2SO_4. Removed the solvents and the crude product was purified by flash column chromatography on silica gel, eluting with 5%MeOH in DCM to afford the desired product as a white solid.

Step b: 2-chloro-*N*-(4-methoxyphenyl)pyrimidine-5-carboxamide (52 mg, 0.20 mmol) and 1-(pyridin-2-yl)piperazine (32 mg, 0.20 mmol) was dissolved in 4 mL dry MeCN. Et_3N (30 mg, 0.30 mmol) was added to the solution. The mixture was stirred at room temperature overnight. Removed the solvent and the crude product was purified by flash column chromatography on silica gel, eluting with hexanes/ethyl acetate (with 5% 7 N NH_3 in MeOH) (4:1 to 1:1) to afford the desired product as a white solid.

2-chloro-*N*-(4-methoxyphenyl)pyrimidine-5-carboxamide (GT-5-110)

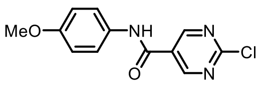

^{1}H NMR (400 MHz, DMSO-*d*$_6$) δ 10.50 (s, 1H), 9.21 (s, 2H), 7.65 (d, *J* = 9.0 Hz, 2H), 6.96 (d, *J* = 9.0 Hz, 2H), 3.75 (s, 3H); **^{13}C NMR (101 MHz, DMSO-*d*$_6$)** δ 162.2, 161.1, 160.1, 156.5, 131.8, 128.1, 122.4, 114.4, 55.7. **LCMS**: 264 $[M+H]^+$. White solid, 101 mg, 19%.

***N*-(4-methoxyphenyl)-2-(4-(pyridin-2-yl)piperazin-1-yl)pyrimidine-5-carboxamide (GT-5-121)**

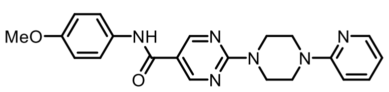

^{1}H NMR (400 MHz, DMSO-*d*$_6$) δ 9.99 (s, 1H), 8.91 (s, 2H), 8.14 (dd, *J* = 4.9, 2.0 Hz, 1H), 7.62 (d, *J* = 9.0 Hz, 2H), 7.59 – 7.54 (m, 1H), 6.92 (d, *J* = 9.0 Hz, 2H), 6.88 (d, *J* = 8.7 Hz, 1H), 6.67 (dd, *J* = 7.1, 4.9 Hz, 1H), 3.96 (t, *J* = 5.3 Hz, 4H), 3.74 (s, 3H), 3.62 (t, *J* = 5.3 Hz, 4H); **^{13}C NMR (101 MHz, DMSO-*d*$_6$)** δ 162.2, 161.4, 158.8, 158.0, 155.5, 147.6, 137.7, 132.0, 121.9, 116.7, 113.8, 113.3, 107.3, 55.2, 44.3, 43.2. **LCMS**: 391 $[M+H]^+$; **HRMS** (ESI) calculated for $C_{21}H_{23}N_6O_2^+$ ($[M+H^+]$) 391.1877, found: 391.1879. White solid, 59 mg, 76%.

Method for the synthesis of GT-2-114

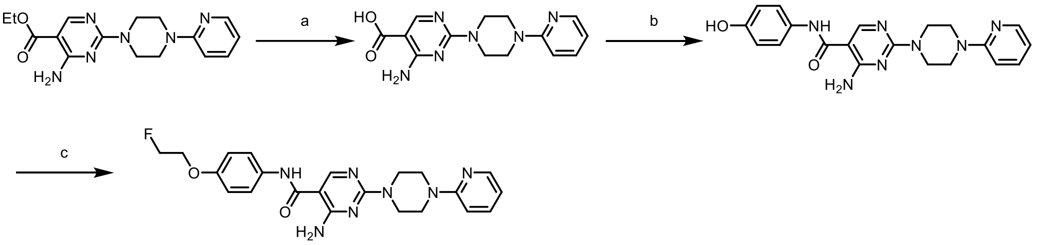

Conditions: a. NaOH, EtOH/H_2O, 60 °C, 4 h; b. 4-Aminophenol, HBTU, DIPEA, DMF, rt. to 50 °C, overnight; c. 1-fluoro-2-iodoethane, Cs_2CO_3, DMF, 60 °C, 6 h.

Step a: **GT-5-46** (0.20 mmol) was dissolved in EtOH and H_2O (2 mL/2 mL). The mixture was heated to 60 °C for 4 h. After cooling to room temperature, EtOH was removed under reduced pressure. Saturated $NaHCO_3$ and 1 N HCl were used to make the solution to PH = 7. The precipitated solid was filtered out and dried on a high vacuum for 12 h. The crude product was used for the next step directly.

Step b: A solution of the crude product above, DIPEA (0.6 mmol) and HBTU (0.21 mmol) in 4 mL DMF was stirred for 15 min. at room temperature. 4-Aminophenol (26 mg, 0.21 mmol) in DMF (1 mL) was added to the solution. The mixture was stirred at room temperature overnight. The mixture was washed with 1 N $NaHCO_3$ solution and extracted with DCM (3× 15 mL) and dried over Na_2SO_4. Removed the solvent and the residue was purified by flash column chromatography on silica gel, eluting with DCM/7 N NH_3 in MeOH = 95: 5 to afford the desired product as a solid.

4-amino-*N*-(4-hydroxyphenyl)-2-(4-(pyridin-2-yl)piperazin-1-yl)pyrimidine-5-carboxamide (GT-5-96)

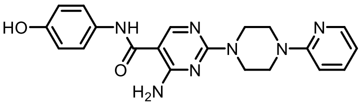

^{1}H NMR (400 MHz, DMSO-*d*$_6$) δ 9.66 (s, 1H), 9.22 (s, 1H), 8.59 (s, 1H), 8.13 (ddd, *J* = 5.0, 2.0, 0.8 Hz, 1H), 7.56 (ddd, *J* = 8.9, 7.1, 2.0 Hz, 1H), 7.39 (d, *J* = 8.9 Hz, 2H), 6.87 (d, *J* = 8.6 Hz, 1H), 6.71 (d, *J* = 8.9 Hz, 2H), 6.66 (ddd, *J* = 7.2, 4.8, 0.8 Hz, 1H), 3.89 – 3.83 (m, 4H), 3.59 – 3.52 (m, 4H); **^{13}C NMR (101 MHz, DMSO-*d*$_6$)** δ 165.2, 162.9, 160.9, 158.9, 157.3, 153.5, 147.6, 137.6, 130.5, 122.6, 114.9, 113.2, 107.3, 99.1, 44.5, 42.9. **LCMS**: 392 $[M+H]^+$. White solid, 41 mg, 53%.

Step c: A solution of **GT-5-96** (0.2 mmol), Cs_2CO_3 (0.4 mmol) and 1-fluoro-2-iodoethane (0.4 mmol) in DMF (4 mL) was heated to 60 °C and stirred for 6 h. The solvents were removed and the crude product was purified by flash column chromatography on silica gel, eluting with DCM/MeOH = 95: 5 to afford the desired product as a solid.

4-amino-*N*-(4-(2-fluoroethoxy)phenyl)-2-(4-(pyridin-2-yl)piperazin-1-yl)pyrimidine-5-carboxamide (GT-2-114)

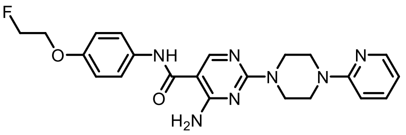

^{1}H NMR (400 MHz, DMSO-*d*$_6$) δ 9.76 (s, 1H), 8.61 (s, 1H), 8.13 (ddd, *J* = 4.9, 2.0, 0.8 Hz, 1H), 7.57 – 7.53 (m, 3H), 6.93 (d, *J* = 9.1 Hz, 2H), 6.87 (d, *J* = 8.6 Hz, 1H), 6.66 (ddd, *J* = 7.1, 4.9, 0.8 Hz, 1H), 4.81 – 4.77 (m, 1H), 4.69 – 4.65 (m, 1H), 4.26 – 4.22 (m, 1H), 4.18 – 4.14 (m, 1H), 3.90 – 3.84 (m, 4H), 3.59 – 3.54 (m, 4H); **^{13}C NMR (101 MHz, DMSO-*d*$_6$)** δ 165.4, 162.9, 160.9, 158.9, 157.5, 154.2, 147.6, 137.6, 132.4, 122.2, 114.4, 113.2, 107.2, 99.0, 82.2 (d, *J* = 166.7 Hz), 67.2 (d, *J* = 19.0 Hz), 44.4, 42.9. **LCMS**: 438 $[M+H]^+$; **HRMS** (ESI) calculated for $C_{22}H_{25}FN_7O_2^+$ ($[M+H^+]$) 438.2048, found: 438.2044. White solid, 30 mg, 68%.

Method for the synthesis of GT-2-123 and related data.

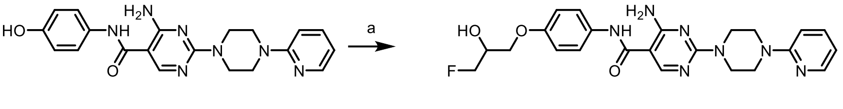

GT-5-96

Conditions: a. 2-(fluoromethyl)oxirane, Cs_2CO_3, DMF, 80 °C, 6 h.

Step a: A solution of **GT-5-96** (39 mg, 0.1 mmol), Cs_2CO_3 (0.2 mmol) and 2-(fluoromethyl)oxirane (0.2 mmol) in DMF (2 mL) was heated to 60 °C and stirred for 6 h. The solvents were removed and the crude product was purified by flash column chromatography on silica gel, eluting with DCM/MeOH = 95: 5 to afford the desired product as a solid.

4-amino-*N*-(4-(3-fluoro-2-hydroxypropoxy)phenyl)-2-(4-(pyridin-2-yl)piperazin-1-yl)pyrimidine-5-carboxamide (GT-2-123)

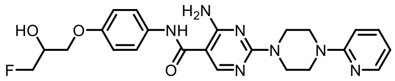

^{1}H NMR (400 MHz, DMSO-*d*$_6$) δ 9.75 (s, 1H), 8.61 (s, 1H), 8.13 (ddd, *J* = 4.9, 2.0, 0.8 Hz, 1H), 7.59 – 7.51 (m, 4H), 6.93 – 6.85 (m, 3H), 6.68 – 6.64 (m, 1H), 5.42 (d, *J* = 5.2 Hz, 1H), 4.59 – 4.38 (m, 2H), 4.04 – 3.93 (m, 3H), 3.90 – 3.83 (m, 4H), 3.59 – 3.52 (m, 4H); **^{13}C NMR (101 MHz, DMSO-*d*$_6$)** δ 165.4, 162.9, 160.9, 158.9, 157.4, 154.5, 147.6, 137.6, 132.3, 122.2, 114.4, 113.2, 107.2, 99.0, 84.4 (d, *J* = 168.2 Hz), 68.3 (d, *J* = 7.9 Hz), 67.7 (d, *J* = 19.0 Hz), 44.4, 42.9.

GT-2-123: LCMS: 468 [M+H]$^+$; **HRMS** (ESI) calculated for $C_{23}H_{27}FN_7O_3^+$ ([M+H$^+$]) 468.2154, found: 468.2155. White solid, 24 mg, 53%.

Methods for the synthesis of GT-2-122 and GT-2-146 and related data.

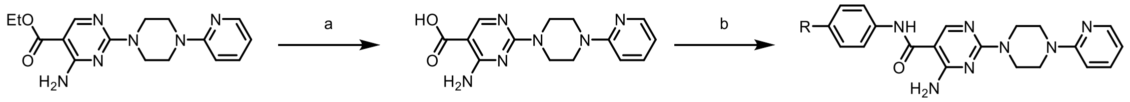

Conditions: a. NaOH, EtOH/H_2O, 60 °C, 4 h; b. 4-fluoroaniline, 4-(trifluoromethyl)aniline, 2-fluoro-4-methoxyaniline or 3-fluoro-4-methoxyaniline, HBTU, DIPEA, DMF, rt. to 50 °C, overnight.

Step a: **GT-5-46** (0.20 mmol) was dissolved in EtOH and H_2O (2 mL/2 mL). The mixture was heated to 60 °C for 4 h. After cooling to room temperature, EtOH was removed under reduced pressure. 1 N HCl was used to make the solution to PH = 7. The precipitated solid was filtered out and dried on a high vacuum for 12 h. The crude product was used for the next step directly.

Step b: A solution of the crude product above, DIPEA (77 mg, 0.6 mmol) and HBTU (0.21 mmol) in DMF (4 mL) was stirred for 15 min. at room temperature. Corresponding amine (0.21 mmol) in DMF (1 mL) was added to the solution. The mixture was heated to 50 °C and stirred overnight. After cooling to room temperature, the mixture was washed with 1 N Na_2CO_3 solution and extracted with DCM (3×15 mL), and dried over Na_2SO_4. The solvent were removed and the residue was purified by flash column chromatography on silica gel, eluting with DCM/7 N NH_3 in MeOH = 95: 5 to afford the desired product as a solid.

4-amino-*N*-(4-fluorophenyl)-2-(4-(pyridin-2-yl)piperazin-1-yl)pyrimidine-5-carboxamide (GT-2-122)

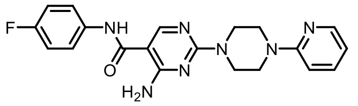

^{1}H NMR (400 MHz, DMSO-*d*$_6$) δ 9.90 (s, 1H), 8.63 (s, 1H), 8.13 (ddd, *J* = 4.9, 2.1, 0.8 Hz, 1H), 7.70 – 7.62 (m, 2H), 7.56 (ddd, *J* = 8.9, 7.1, 2.0 Hz, 2H), 7.19 – 7.11 (m, 2H), 6.87 (d, *J* = 8.6 Hz, 1H), 6.66 (ddd, *J* = 7.1, 4.8, 0.8 Hz, 1H), 3.94 – 3.82 (m, 4H), 3.60 – 3.51 (m, 4H); **^{13}C NMR (101 MHz, DMSO-*d*$_6$)** δ 165.6, 162.9, 160.9, 158.9, 158.1 (d, *J* = 241.9 Hz), 157.7, 147.6, 137.6, 135.4, 122.4 (d, *J* =

7.8 Hz), 115.1 (d, *J* = 22.1 Hz), 113.2, 107.2, 98.8, 44.4, 42.9. **LCMS**: 394 [M+H]$^+$; **HRMS** (ESI) calculated for $C_{20}H_{21}FN_7O^+$ ([M+H$^+$]) 394.1786, found: 394.1782. White solid, 28 mg, 36%.

4-amino-2-(4-(pyridin-2-yl)piperazin-1-yl)-*N*-(4-(trifluoromethyl)phenyl)pyrimidine-5-carboxamide (GT-2-146)

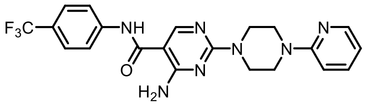

^{1}H NMR (400 MHz, DMSO-*d*$_6$) δ 10.16 (s, 1H), 8.68 (s, 1H), 8.18 – 8.12 (m, 2H), 7.90 (d, *J* = 8.5 Hz, 2H), 7.69 (d, *J* = 8.6 Hz, 2H), 7.61 – 7.55 (m, 3H), 6.90 – 6.86 (m, 2H), 6.70 – 6.66 (m, 2H), 3.90 (t, *J* = 5.4 Hz, 4H), 3.58 (t, *J* = 5.4 Hz, 4H); **^{13}C NMR (101 MHz, DMSO-*d*$_6$)** δ 166.5, 163.4, 159.4, 158.6, 148.1, 138.1, 126.3, 126.3, 120.5, 113.7, 107.7, 99.1, 44.9, 43.4. **LCMS**: 444 [M+H]$^+$; **HRMS** (ESI) calculated for $C_{21}H_{21}F_3N_7O^+$ ([M+H$^+$]) 444.1754, found: 444.1755. Light-yellow solid, 5 mg, 5%.

Methods for the synthesis of GT-2-149 and related data.

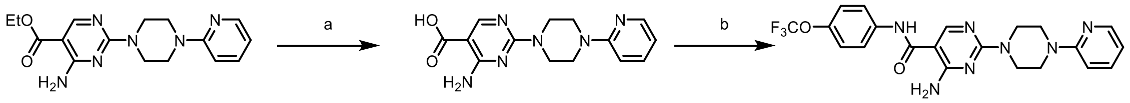

Conditions: a. NaOH, EtOH/H_2O, 60 °C, 4 h; b. 4-(trifluoromethoxy)aniline, EDCl•HCl, DMAP, Et_3N, DMF, rt. to 50 °C, overnight.

Step a: **GT-5-46** (0.20 mmol) was dissolved in EtOH and H_2O (2 mL/2 mL). The mixture was heated to 60 °C for 4 h. After cooling to room temperature, EtOH was removed under reduced pressure. 1 N HCl was used to make the solution to PH = 7. The precipitated solid was filtered out and dried on a high vacuum for 12 h. The crude product was used for the next step directly.

Step b: A solution of the crude product above, Et_3N (0.6 mmol) and DMAP (0.02 mmol), and EDCI•HCl (0.21 mmol) in DMF (4 mL) was stirred for 15 min. at room temperature. 4-(trifluoromethoxy)aniline (0.21 mmol) in DMF (1 mL) was added to the solution. The mixture was heated to 50 °C and stirred overnight. After cooling to room temperature, the mixture was washed with 1 N Na_2CO_3 solution and extracted with DCM (3×15 mL) and dried over Na_2SO_4. Removed the solvent and the residue was purified by flash column chromatography on silica gel, eluting with DCM/7 N NH_3 in MeOH = 95: 5 to afford the desired product as a solid.

4-amino-2-(4-(pyridin-2-yl)piperazin-1-yl)-*N*-(4-(trifluoromethoxy)phenyl)pyrimidine-5-carboxamide (GT-2-149)

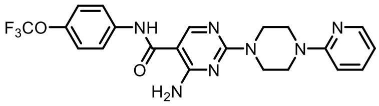

^{1}H NMR (400 MHz, DMSO-*d*$_6$) δ 10.02 (s, 1H), 8.64 (s, 1H), 8.13 (dd, *J* = 5.0, 2.0 Hz, 1H), 7.76 (d, *J* = 9.1 Hz, 2H), 7.56 (ddd, *J* = 8.9, 7.0, 2.0 Hz, 2H), 7.32 (d, *J* = 8.6 Hz, 2H), 6.87 (d, *J* = 8.6 Hz, 1H), 6.66 (dd, *J* = 7.1, 4.8 Hz, 1H), 3.88 (dd, *J* = 6.9, 3.8 Hz, 4H), 3.57 (dd, *J* = 6.4, 3.9 Hz, 4H); **^{13}C NMR (101 MHz, DMSO-*d*$_6$)** δ 166.2, 163.4, 161.4, 159.4, 158.4, 148.0, 144.1, 138.9, 138.1, 122.2, 121.8, 119.4, 113.7, 107.7, 99.2, 44.9, 43.4. **LCMS**: 460 [M+H]$^+$; **HRMS** (ESI) calculated for $C_{21}H_{21}F_3N_7O_2^+$ ([M+H$^+$]) 460.1703, found: 460.1711.Light-yellow solid, 32 mg, 36% for 2 steps.
